# New alternatives to holder pasteurization in processing donor milk in human milk banks

**DOI:** 10.3389/fnut.2024.1409381

**Published:** 2024-06-26

**Authors:** Guido E. Moro, Melissa Girard, Chiara Peila, Nadia Garcia, Diana Escuder-Vieco, Kristin Keller, Tanya Cassidy, Enrico Bertino, Clair-Yves Boquien, Rachel Buffin, Javier Calvo, Antoni Gaya, Corinna Gebauer, Delphine Lamireau, David Lembo, Jean-Charles Picaud, Aleksandra Wesolowska, Sertac Arslanoglu, Laura Cavallarin, Marzia Giribaldi

**Affiliations:** ^1^Associazione Italiana delle Banche del Latte Umano Donato (AIBLUD), Milan, Italy; ^2^Medical Affairs and Innovation, Héma-Québec, Québec, QC, Canada; ^3^Neonatal Unit, Department of Public Health and Pediatrics, University of Turin, Turin, Italy; ^4^Banco Regional de Leche Materna Aladina-MGU, Hospital Universitario 12 de Octubre, Madrid, Spain; ^5^Kathleen Lonsdale Health Research Institute, Maynooth University, Maynooth, Ireland; ^6^Nantes Université, INRAE, UMR 1280, PhAN, CRNH-OUEST, Nantes, France; ^7^Neonatology UnitCroix-Rousse University Hospital, Hospices Civils de Lyon, Lyon, France; ^8^Rhône-Alpes-Auvergne Regional Human Milk Bank, Croix-Rousse University Hospital, Hospices Civils de Lyon, Lyon, France; ^9^Group of Cell Therapy and Tissue Engineering (TERCIT), Fundació Banc de Sang i Teixits de les Illes Balears (FBSTIB), Research Institute on Health Sciences (IUNICS) and Health Research Institute of the Balearic Islands (IdISBa), Palma, Spain; ^10^Abteilung Neonatologie Klinik und Poliklinik für Kinder und Jugendliche, Leipzig, Germany; ^11^Human Milk Bank of University Hospital of Bordeaux, Lamireau, France; ^12^Laboratory of Molecular Virology and Antiviral Research, Department of Clinical and Biological Sciences, University of Turin, Turin, Italy; ^13^Department of Neonatology, Hôpital de la Croix-Rousse, Hospices Civils de Lyon, Lyon, France; ^14^CarMen Laboratory, INSERM, INRA, Université Claude Bernard Lyon1, Lyon, France; ^15^Laboratory of Human Milk and Lactation Research at Milk Bank in Holy Family Hospital, Department of Medical Biology, Faculty of Health Science, Medical University of Warsaw, Warsaw, Poland; ^16^Division of Neonatology, Department of Pediatrics, Istanbul Medeniyet University, Istanbul, Türkiye; ^17^Consiglio Nazionale delle Ricerche, Istituto di Scienze delle Produzioni Alimentari, Turin, Italy

**Keywords:** human milk, donor human milk, human milk bank, HTST, HPP

## Abstract

Infectious and toxicological risks are the main potential hazards that operators of Human Milk Banks (HMBs) encounter and must eliminate. HMBs are trying to implement procedures that allow to manage and sanitize human milk without altering significantly its nutritional and biologically protective components, obtaining a product characterized by a valid balance between safety and biological quality. The history of human milk processing is linked to the origins of HMBs themselves. And although other forms of sterilization were used originally, pasteurization soon became the recognized most effective means for sanitizing milk: all the milk that arrives at the HMB must be pasteurized. Holder pasteurization (HoP) is the most used methodology, and it is performed using low temperature and long time (+62.5°C for 30 min). With HoP some bioactive milk components are lost to varying degrees, but many other precious bioactive compounds are completely or partially preserved. To improve the quality of human milk processed by HMBs, maintaining in the meantime the same microbiological safety offered by HoP, new technologies are under evaluation. At present, High-Temperature Short-Time pasteurization (HTST) and High-Pressure Processing are the most studied methodologies. HTST is already utilized in some HMBs for daily practical activity and for research purposes. They seem to be superior to HoP for a better preservation of some nutritional and biologically protective components. Freeze-drying or lyophilization may have advantages for room temperature storage and transportation. The aim of this study is to evaluate the advancement regarding the processing of DHM with a literature search from 2019 to 2022. The effects of the new technologies on safety and quality of human milk are presented and discussed. The new technologies should assure microbiological safety of the final product at least at the same level as optimized HoP, with an improved preservation of the nutritional and bioactive components of raw human milk.

## 1 Introduction

Holder pasteurization (HoP) is the processing method most often recommended in relevant guidelines around the world (1). For this paper, we are including some information about the historical origins of processing of human milk (HM) in donor human milk (DHM) banks. We can always learn from the past, although each of the specific processing methods we are going to consider (some of which we also discussed previously) have a history of their own. We begin this paper with a discussion of the original processing method conducted in Vienna in 1909.

It has been recognized that the origins of milk banking itself are derived from the scientific advancement of HM processing, and to the laboratories in Vienna in 1909 linked to the clinical biologist Theodor Escherich (1–4). The first DHM service in Vienna did not use pasteurization, but instead used the most popular European method for sterilization at the time, known as the “Budde” method, named after Carl Christian Leopold Getter (C.C.L.G) Budde from Denmark, and based on original experiments from 1906 which were first published in 1908. The Budde method became the state-of-the-art process and was increasingly popular across Europe (5). It had previously been reported on in *Scientific American* in 1903 (6). The Budde method includes heating at a low temperature, but it also involves the addition of hydric dioxide to sterilize the milk. This obsolete term “buddeization”, meaning to sterilize milk, was widely used at least until World War II, as is evidenced by a 1937 American thesis which compared this method to milk “iodization” (milk treatment by iodine) and considered the effects of these two processes on enzymes, bacterial content, physical and chemical properties, and nutritional value of milk (7).

In light of limited refrigeration, preservation was a driving concern, and this was especially true during the summer months when spoilage occurred much more quickly, and these concerns were linked to the commercial pasteurization of bovine milk. The history of pasteurizing milk in bottles while heating dates to the 1890s (8, 9). The “in bottle” process of heating to 62.8°C (145°F) for 20–30 min, which also has been argued to date from the early 1900s, whereas the so-called commercial use of the “holder” or “in vat” method of pasteurization at a similar temperature for a similar length of time is argued to have become common, especially in large urban centers, by 1917 (9, 10), although used commercially earlier. It is around this time that we start to see this method become more common when pasteurizing HM as well, at least the in-bottle version, beginning it seems in US urban centers (11), in particular New York (12).

High-temperature-short-time (HTST) pasteurization, also known as “flash” pasteurization, was also conducted in the US in the early part of the twentieth century, but was not considered appropriate and abandoned until the 1920s (9, 10). High pressure processing or high hydrostatic pressure processing (HPP), also known as “pascalization” or “bridgmanization,” also dates to the end of the nineteenth century (13, 14) but is not used more widely or with HM until much more recently (15). This is likewise the case with short wave ultraviolet (UV) irradiation (16). On the other hand, the history of lyophilization or freeze drying of HM for their conservation could be traced back to the 1920s (17, 18) and is tied to the service at the so-called Boston Floating hospital and Massachusetts Institute of Technology-MIT. Although, each of these processes has its own history, it becomes clear that even some of the most recent processes have been part of the scientific imagination for a very long time.

## 2 Aim of the study

In most human milk banks (HMBs) worldwide, milk processing includes HoP, which has a significant effect on nutrient content and biological properties of DHM. In a recently published paper on this topic by the European Association of Human Milk Banks (EMBA) (19), the conclusions were that in processing of DHM new technologies are developing rapidly, and EMBA recommendations were that “…*the final aim of these technologies should be an improved preservation of the nutritional and bioactive components of raw human milk, while assuring microbiological safety of the product, at least at the same level of optimized HoP*.”

This aspect is considered by EMBA as a scientific priority in the field of HM donation and processing in HMBs. Therefore, the EMBA Board of Directors set up a Working Group (WG) in the year 2018 with the purpose to perform research in this field. The components of this WG are relevant scientists, from different European countries with a great experience in the field of HMBs and HM treatment. The first step of this WG was to update the knowledge about the technologies most utilized in HMBs and to come out with recommendations based on the results of this search and on their personal experience (19). The second step is the present study, aimed to evaluate the state of the art today, with a literature search from 2019 (year of publication of the first EMBA paper on processing of DHM) to the end of 2022. The purpose is to evaluate the new most advanced technologies of HM treatment, and to compare, when possible, the results obtained from these technologies with HoP. Review articles were excluded as well as conference paper/abstract, book chapters, and position paper.

## 3 High-temperature short-time (HTST) pasteurization

HTST (generally 72°C for 15 s) is a thermal sanification technology whose application in dairy and beverage industry dates to the beginning of XX century. Since the publication of the last version of EMBA recommendations (19), the number of reports on the efficacy and effect of HTST treatment applied to HMBs environment has increased. Some of the improvement points previously listed by EMBA working group have been further addressed, concerning instrumental and processing conditions and validation in HMB environment. Also, studies on the inactivation of HM viruses by HTST pasteurizers have been carried out in recent years. It is important to underline that HTST efficacy against viruses was previously investigated at laboratory level by two research teams (20, 21), whose work contributed to the design of HTST devices that are still being used nowadays (22–24), also in the clinical practice (25, 26).

Briefly, a literature review has been conducted by searching separately the following terms in the Title/Abstract/Keywords domains in Scopus, and in Title/Abstract/MeSH terms in PubMed, limiting the search in the time range 2019–2022: (“human milk” OR “donor milk” OR “breastmilk”) AND (“High – Temperature – Short – Time” OR “High Temperature Short Time” OR “HTST” OR “Flash pasteurization” OR “Flash pasteurization”). Results retrieved from single database were: 27 in Scopus and 106 in PubMed. After filtering by 2 independent authors (Ma.G. and C.Y.B.), a total of 17 papers were considered as relevant for the present review.

### 3.1 HTST results

Tables 1–3 summarize the reports that have been found from 2019 to 2022 on the use of HTST processing for treating donor HM. The most relevant point is that the number of patented and/or commercially available instruments has increased, with respect to the two prototypes already described in 2018 (Spanish patented prototype – PCT/ES2016/070594; Italian patented prototype –EP 15176792.8–1358). Nevertheless, literature search still reports experiments that simulated HTST processing by means of submerging bottles/tubes for few seconds in hot water (27, 29), or by using small volumes, like polymerase chain reaction (PCR) tubes (34). These processes do not meet the requirements of repeatability and monitoring of the temperatures that are needed for implementation in HMB environment. Another aspect is that different groups reported results achieved by different time/temperature combinations. Although these results can be interesting to optimize the processing parameters of HTST, and to limit the thermal damage of DHM components, the pre-requisite for testing different processing conditions should be that these combinations using the same equipment have been already proved as effective as HoP in eradicating bacterial and viral pathogens.

**Table 1:** Effects of HTST on safety of human milk.*

	References	HTST treatment parameters	Device	Milk components/properties	Impact of treatment (compared to raw milk)	Comparison HTST – HoP
*Pathogens* Microbicidal/Antimicrobial activity	Capriati et al. (27)	Fixed time/temperature: 70°C for few min. (not specified) pasteurized volume: 30 mL each batch *Donors n = 2*	Submerged bottles in a HoP pasteuriser modified to reach the determined plateau temperature.	Microbiological counts of native milk microflora (Total aerobic, *Staphylococcus aureus*, *Bacillus cereus*)	Undetectable after treatment (except *B. cereus* – one sample)	Similar eradication in HoP
	Aceti et al. (28)	Fixed time/temperature: 72°C for 15 s pasteurized volume total: 100 mL *Donors n = 5*	Patented prototype (EP 15176792.8–1,358).	Microbiological counts of native milk microflora (Total aerobic, *Staphylococcus aureus*, Enterobacteriaceae)	Undetectable microbial counts after treatment	Similar eradication in HoP
	Manzardo et al. (26)	Different time/ temperature combinations: 62 and 81°C for 5 s. pasteurized volume 60 mL each batch *Donors n = 30*	Virex II (Lauf, Tübingen, Germany)	Efficacy against inoculated microorganisms	Combination 81°C/5 s. resulted in no bacterial growth, with the exception of *E. faecalis*	HoP: 5 log reduction for all microorganisms except *C. sakazakii* (RV00078)
				*Enterococcus faecalis* ATC 29212–5.8 × 10^4^ CFU/mL	62°C for 5 s: 0.76 log reduction; 81°C for 5 s: 3.3 log reduction	
				*Cronobacter sakazakii* RV00078 and RV5-I-92 – 1.4 and 2.1 × 10^5^ CFU/mL	62°C for 5 s: 1.9 and 1.3 log reduction; 81°C for 5 s: 5 log reduction	
				*Listeria monocytogenes* 00218 and 0015–1.8 and 1.1 × 10^5^ CFU/mL	62°C for 5 s: 1.2 and 1 log reduction; 81°C for 5 s: 5 log reduction	
				Cytomegalovirus (HCMV)	As effective as raw human milk	
				Respiratory syncytial virus (RSV)	As effective as raw human milk	
				Rotavirus (HRoV)	As effective as raw human milk	
				Rhinovirus (HRhV)	Reduced inhibitory dilution (HTST 0.06 vs. Raw 0.02)	
				Herpes simplex virus type 1 and 2 (HSV-1, HSV-2)	As effective as raw human milk against HSV-2; reduced inhibitory dilution against HSV-1 (HTST 0.08 vs. Raw 0.05)	
	Patil et al. (29)	Fixed time/temperature: 72°C for 15 s. pasteurized volume: 10 mL each batch *Donors n = 48, pooled*	Submerged bottles in a HoP pasteuriser modified to reach the determined plateau temperature:	Bactericidal activity against *E. coli*	*Reduction in bacterial growth:* after 4 h incubation: 95% after 8 h incubation: 94% after 24 h incubation: 26%	HoP showed a higher *E. coli* bactericidal activity than HTST after 24 h incubation (46%)
	Kontopodi et al. (24)	Fixed time/temperature: 72°C for 15 s. pasteurized volume total: 50 mL *Donors n = 4, pooled*	Laboratory scale pasteuriser	Bacteriostatic activity against *E. coli* and *S. aureus*	*Growth rate per hour:* significant increase in *E. coli* growth rate (HTST 4.2 vs. Raw 3.6), but similar for *S. areus* (growth rate 2.0 -fold per hour)	HoP caused the highest reduction in bacteriostatic capacity.

*p-value are indicated only when significant and/or reported in the original article; NS, not significantly different.

**Table 2:** Effects of HTST on quality of human milk: nutrients and bioactive factors retention.*

	Reference	HTST treatment parameters	Device	Milk components/properties	Impact of treatment (compared to raw milk)	Comparison HTST – HoP
Nutrients	Capriati et al. (27)	Fixed time/temperature: 70°C for few min. pasteurized volume: 30 mL each batch *Donors n = 2*	Submerged bottles in a HoP pasteuriser modified to reach the determined plateau temperature	Calcium (mg%)	Not affected	HoP showed similar results, with the exception of slightly higher triglycerides decrease (~21%, not significant)
				Amino acids profile (mcgmol/L)	Not affected	
				Triglycerides (mg%)	Decreased (~18%)	
	Aceti et al. (28)	Fixed time/temperature: 72°C for 15 s pasteurized volume total: 100 mL *Donors n* = 5	Patented prototype (EP 15176792.8–1,358)	Total protein content	Not affected	HoP showed similar results
	Escuder-Vieco et al. (30)	Different time/temperature combinations: 70, 72, 75°C for 5, 10, 15, 20, and 25 s. Pasteurized volume total: 9760 mL each batch *Donors n = 12, pooled in each batch*	Patented prototype – (PCT/ES2016/070594)	Total protein content (g/L)	Not affected	HoP preserved the fat and lactose content, myo -inositol and vitamins decreased the total protein content (−1%); pyridoxal (−12%) - increased the glucose (+8%); vitamin D3 (+47%).
				Total fat content (g/L)	Not affected at 5–15 s; −4% at 25 s.	
				Lactose content (g/L)	Slightly increased (<1%)	
				Glucose content (mg/L)	Not affected until 20 s.; −10% at 25 s.	
				Myo – inositol content (mg/L)	Not affected	
				Vitamin content	Not affected	
				Lipid profile	Similar triglyceride content Higher diglycerides at 5–10 s. Lower (~50%) mono-glycerides Doubled phospholipid fraction 1/3 reduced cholesterol and free fatty acid Minor significant reduction of Saturated Fatty Acids (−5%), and increase in Polyunsaturated Fatty Acids (+7%) Duration rather than temperature seemed to have an impact on nutrients	
Bioactive factors	Aceti et al. (28)	Fixed time/temperature: 72°C for 15 s pasteurized volume total: 100 mL *Donors n* = 5	Patented prototype (EP 15176792.8–1,358)	Lactoferrin content	Decreased (−83.5%)	HoP shows a similar retention of lactoferrin content and sIgAs content. The protein profile revealed aggregation phenomena for native lactoferrin that was significantly reduced by HoP (−35%)
				Secretory IgA content	Preserved (−20%, not significant)	
				Protein denaturation (PAGE)	Protein profile analysis revealed an induction of protein aggregation (>40%), and a decrease of native lactoferrin band (although not significant)	
	Escuder-Vieco et al. (30)	Different time/temperature combinations: 70, 72, 75°C for 5, 10, 15, 20, and 25 s. pasteurized volume total: 9760 mL each batch *Donors n = 12, pooled in each batch*	Patented prototype – (PCT/ES2016/070594)	Bile salt stimulated lipase activity (%)	Mean retention <20%	Lower retention by HoP (7%).
	Kontopodi et al. (24)	Fixed time/temperature: 72°C for 15 s. pasteurized volume total: 50 mL *Donors n = 4, pooled*	Laboratory scale pasteuriser.	Native serum protein content (mg/ml)	Not affected	HoP caused a lower retention than HTST for all proteins studied (~50% serum proteins; 44% IgAs; 19% lactoferrin; 2% BSSL concentration and 4% activity), except lysozyme, and alkaline phosphatase (not significant)
				IgAs concentration	Not affected	
				Lysozyme concentration	Not affected	
				Lactoferrin concentration	Decreased (~50%)	
				Bile salt-stimulated lipase concentration and activity (%)	Decreased (−91% concentration and −81% activity)	
				Alkaline phosphatase activity (U/ml)	No detectable activity	
	Mank et al. (23)	Fixed time/temperature: 72°C for 15 s. pasteurized volume: 10 mL *Donors n* = 28	Laboratory scale pasteuriser	Insulin concentration (pmol/L)	Not affected (retention 78%)	HoP significantly reduced insulin concentration (retention 67%)
	Manzardo et al. (26)	Different time/temperature combinations: 62°C for 5 s. 72°C for 5 s. 81°C for 5 s. Pasteurized volume: 60 mL *Donors* n = 10	Virex II (Lauf, Tübingen, Germany)	Alkaline phosphatase activity (mU/ml)	Not detected at 72 and 81°C	HoP caused a lower retention than HTST at 62°C, but similar at higher temperatures, except for sIgAs, that were more preserved by HoP and HTST at 62°C.
				Lactoferrin concentration (g/L)	−50% at 62°C, −75 to 80% at 72–81°C	
				Secretory IgA concentration (g/L)	−20% at 62°C, −70% at 72°C, undetectable at 81°C	
	Müller et al. (31)	Fixed time/temperature: 62°C for 5 s. Pasteurized volume: 60 mL *Donors n = 15*	Virex II (Lauf, Tübingen, Germany)	Alkaline phosphatase activity (mU/ml)	Decreased (−50%)	HoP caused a lower retention than HTST for secretory IgAs, lactoferrin, and alkaline phosphatase, in particular when using dry tempering devices.
				Lactoferrin concentration (g/L)	Decreased (−20%)	
				Secretory IgA concentration (g/L)	Decreased (−30%)	

*p-value are indicated only when significant and/or reported in the original article; NS, not significantly different.

**Table 3:** *In vivo*/*In vitro* metabolic studies on HTST effect.*

	References	HTST treatment parameters	Device	Studied milk components/properties	Impact of treatment (compared to raw milk)	Comparison HTST – HoP
*In vitro*	Nebbia et al. (32)	Fixed time/temperature: 72°C for 15 s Pasteurized volume total: 100 mL *Donors* n = 5	Patented prototype (EP 15176792.8–1,358)	Macronutrient composition, protein profiling, confocal microscopy, dynamic light scattering and aminoacid composition of human milk following *in vitro* simulated dynamic digestion with preterm infant model.	Formation of protein aggregates mainly composed by lactoferrin, xanthine dehydrogenase, and fatty acid synthase; 75% retention of native lactoferrin content. Formation of smaller particles during gastric digestion; increased rate of digestion for some protein fractions. Similar lipid composition, and release of free NH2 during digestion.	HoP increased the interaction between proteins and fat globules, decreased the retention of native lactoferrin (33%), and showed a lower abundance of native BSSL than in HTST (−66% vs. −39%). Digestion patterns were similar for proteins (a slightly higher retention of immunoglobulins and lactoferrin, and a higher intestinal release of total and essential free amino acids was seen after HTST).
	Giribaldi et al. (33)	Fixed time/temperature: 72°C for 15 s pasteurized volume total: 100 mL *Donors n* = 5	Patented prototype (EP 15176792.8–1,358)	Peptidomic analysis of *in vitro* simulated dynamic digestion with preterm infant model.	A slightly more similar pattern in peptide release during gastric digestion was found between HTST and raw human milk, in particular with peptide release from beta casein digestion.	During gastric digestion, HOP-HM presented a greater number and more abundant specific peptides from beta casein.
	Hu et al. (22)	Fixed time/temperature: 72°C for 15 s. pasteurized volume total: 10 mL *Donors* n = 30	Laboratory scale pasteuriser	Blood plasma clotting time	Significantly increased >10 fold	HoP significantly increase more than HTST plasma clotting time (>20 fold)

*p-value are indicated only when significant and/or reported in the original article; NS, not significantly different.

#### 3.1.1 Effect on safety of human milk

The effects of HTST on safety of human milk are summarized on Table 1. *S*everal reports on the eradication of bacteria, as well as viruses (mainly CMV), have been published (19, 20, 35). Evidence on the efficacy of HTST on HM microflora is now available for every commercial system (26, 28). The pathogens showing higher resistance to thermal treatments, such as *Enterococcus faecalis*, required temperatures above 72°C, and were more effectively reduced by HoP (26). *Bacillus cereus* was not eradicated even in the most challenging conditions tested, and its presence in DHM is not efficiently resolved by thermal pasteurization alone, requiring a multi-step integrated approach consisting in donor education, monitoring milk management at home, and the instauration of a routine analysis of pasteurized donor milk (36), and a specific detection system in HMBs.

Another aspect that was investigated is the residual bactericidal/bacteriostatic activity of DHM after pasteurization with either HoP or HTST. While the raw HM bacteriostatic activity against *Staphylococcus aureus* was preserved by HTST, as opposite to HoP (24), the bactericidal/bacteriostatic activity against *Escherichia coli* was more reduced over incubation time when HTST was simulated in a modified HoP pasteurizer (29), than by standard HoP. Although a reduction in *E. coli* bacteriostatic activity was reported also by performing HTST in a flow-type laboratory scale system (24), in this case, HTST proved to be more similar than HoP to the native HM bacteriostatic activity, thus demonstrating that the nature of the pasteurization device may have an influence on the HTST performances.

Endospore-forming spoilage bacteria (*Bacillus* spp. and *Paenibacillus* spp.) are the main biological barrier to extend the shelf life of HTST milk. After a storage at 6°C for 21 days, *Bacillus* and *Paenibacillus* counts (when present) were higher in cow’s milk as the temperature of the HTST treatment increased from 72 to 85°C, remaining <2 log_10_ CFU/mL for the first 7 weeks of cold storage (37, 38). So far, there are no specific works dealing with the effect of HTST treatments on the spore-forming bacterial populations of human milk stored at frozen temperatures, but work is in progress to fill this knowledge gap.

#### 3.1.2 Effect on quality of HM

The amount of research data concerning the effect of HTST technology on nutritional and bioactive compounds in HM has increased significantly since the previous edition of EMBA paper (19) (Table 2). The most interesting aspect of those additional data is that they enforce the opinion that batch and continuous processes performed with commercial devices often result in different degrees of thermal damage, affecting the protein fraction. Overall, HTST performed by continuous devices is reported to not affect the total protein content (24, 28, 30), nor the total amino acid profile (39), although protein denaturation and aggregation have been reported (35). Denaturation and aggregation are highly dependent on the applied time/temperature conditions, and, as observed recently by Escuder-Vieco et al. (30), duration, rather than temperature, seems to affect the degradation process of nutrients. They observed that, as compared to raw HM, the lactose concentration was slightly higher in HTST-treated samples, except for longest tested processing time (25 s). Also, glucose concentration in HTST-treated samples was only affected by 25 s treatments. Myo-inositol was not affected by any HTST condition. The fat content after HTST treatments for 5–15 s was not affected, but longer treatments showed lower levels of fat. Compared to raw milk, the content in triglycerides was similar, while diglycerides increased after 5–10 s treatments, showing a concurrent decrease in monoglycerides (about −50%). The phospholipid fraction doubled in proportion (but no difference in the levels of the individual phospholipid was found). The cholesterol fraction and free fatty acids concentrations were both reduced after all HTST treatments. HTST treatment for 15–25 s resulted in lower saturated fatty acids concentrations (−5%), higher proportion of polyunsaturated fatty acids (PUFA) (+7%) and similar monounsaturated fatty acids. HTST treatment did not affect the concentration of any water-or lipid-soluble vitamins (30). Neither HTST nor HoP treatments had a significant effect on the concentration of the Human Milk Oligosaccharide (HMO) Disialyllacto-N-tetraose (34), calcium and amino acid concentrations (27), while insulin content was better retained by HTST than by HoP method (23).

Many recent investigations focused on the possible modifications in the amount or bioactivity of single HM proteins following HTST pasteurization. Alkaline phosphatase activity was suppressed by both continuous (24, 39) and batch (26) processes, at temperatures above 72°C, while activity was partially retained by using mild conditions (62°C, 5 s) (31). Also, bile salt stimulated activity was highly affected by HTST (20% retention rate after 5 s), although to a lower extent than HoP (retention 7%) (24, 30). Lactoferrin concentration is also affected by HTST in a time/temperature correlation depending on manner by both continuous (24, 28) and batch processes (26, 31), as a consequence of aggregation phenomena (28).

Major difference in the performance between batch and continuous devices was observed in the retention of important immunological factors, namely immunoglobulins. Immunoglobulin A (IgA) retention rates with continuous devices were all above 80% (24, 28), better than standard HoP technique, while batch methods showed much lower retention rates (about 30%) at 72°C (26). Escuder-Vieco and colleagues also demonstrated a higher retention for other HM immunoglobulins after HTST pasteurization with respect to HoP, IgG showing the highest preservation rate (99%), followed by IgM (60%), at a combination of 72°C for 10 s (39).

#### 3.1.3 Effect demonstrated *in vivo* and *in vitro* models

The effects of HTST *in vivo* and *in vitro* models are reported on Table 3. HTST was the first investigated HoP alternative to be addressed for possible differences in donor HM digestion with respect to raw DHM (32, 33). By performing simulated *in vitro* dynamic digestion using preterm infant conditions, HTST treatment resulted in a higher retention of immunoglobulins and lactoferrin content, and higher intestinal release of total and essential free amino acids, with respect to HoP (32). Both thermal pasteurization methods were recently found to affect the ability of HM to induce blood plasma clotting, probably because of protein denaturation over the surface of extracellular vesicles (22), although the *in vivo* role of this ability has not been clarified.

### 3.2 HTST discussion

Currently, two different HTST pasteurizers, specifically designed and validated for HM treatment, are available. One batch device is inspired by the pioneering work by Hamprecht and is used mainly as a cytomegalovirus (CMV) limiting tool in Germany (40), at lower time temperature combinations. Due to its low operating volumes (approximately 50–95 mL), it is suitable for the pasteurization of milk from single donation of single mothers in neonatal units. One continuous patented device has been used since 2020 in the Regional HMB, Madrid, Spain, in clinical routine (30, 39). The validation procedure of the device demonstrated that a processing treatment including a combination of 72°C for at least 10 s is efficient in killing any vegetative cell initially present in raw HM samples, including Gram negative and Gram-positive bacteria, yeasts, at microbial counts up to 5.77 log_10_, except for the spore forming *B. cereus*, similarly to HoP.

HTST treatment resulted in better preservation of the nutritional quality of DHM compared to HoP because relevant thermosensitive components (phospholipids, PUFAs, and BSSL – Bile Salt-Stimulated Lipase) were less affected. Furthermore, vitamins did not present a significant reduction after both HTST pasteurization and HoP (30, 39).

Recent evidence for continuous devices confirms that HTST pasteurization of HM allows a higher retention of bioactive factors, such as immunoglobulins, including sIgA, lactoferrin, and BSSL, when compared to HoP. This is very likely to be at the basis of the antiviral activity of HTST treated HM, that, unlike HoP, retained the original HM inhibitory activity against a set of viruses of clinical relevance in infants, as demonstrated for another commercially available continuous pasteurizer (41). Moreover, bacteriostatic activity against *E. coli* and *S. aureus* was less affected than by HoP (24). The degree of thermal damage for specific bioactive proteins, such as immunoglobulins, is more relevant when batch processes are used, and may thus affect the anti-infectious capacities (26, 29).

Last, the closer biochemical profile of HTST and raw HM resulted in a similar peptide digestion profile at gastric level and in higher release of total and essential amino acids at intestinal level from HTST treated HM, with respect to HoP, as reported by simulated preterm infant digestion (32, 33).

## 4 High pressure processing (HPP)

HPP is used since several years in the food industry to increase the safety and to improve the shelf-life of different products. HPP is usually performed, as a non-thermal pasteurization method, by applying high hydrostatic pressure, from 350 to 800 MPa, for a short period of time (5–10 min). The first review on HM processing by EMBA in 2019 (19) showed promising results when HPP was applied for the treatment of DHM. Scientific teams have demonstrated the destruction of multiple vegetative bacteria, as well as sporulating bacteria, and a better retention of immunoglobulins, lysozyme, lactoferrin, lipase, and cytokines in HPP-treated milk, compared to milk pasteurized by HoP. Given these data, HPP currently experience an increased interest in research on HM and other recent advancements have been made using different HPP settings.

Briefly, searches on Pubmed and Scopus databases have been conducted by using the following terms: (“human milk” OR “donor milk” OR “breastmilk”) AND (“high-pressure processing” OR “high hydrostatic pressure”), limiting the search in the time range 2019–2022. After the screening of 37 publications for eligibility, a total of 23 papers were used for the present review.

### 4.1 HPP results

Tables 4–6 report the most relevant data that have been found in literature on the use of HPP processing for the treatment of DHM.

**Table 4:** Effects of HPP on safety of human milk*.

	Reference	HPP treatment parameters	Device	Studied milk components/properties	Impact of treatment (compared to raw milk)	Comparison HoP – HPP
Pathogens	Kontopodi et al. (24)	Many conditions have been tested: −400 MPa 5, 10, 30 min −500 MPa 1.5, 2 × 1.5, 3, 5 min −600 MPa 1.5, 2 × 1.5, 3, 5 min (*n* = 2)	Pilot-scale equipment (Resato, Roden, Netherlands).	Inactivation of *E. cloacae* and *S. aureus*	>7.8 Log_10_ reduction (below detection limit) for all HPP-tested parameters	The same inactivation was obtained after HoP for all tested bacterial strains. (>7.8 Log_10_ reduction; below detection limit).
				Bacteriostatic capacity	NS	NS
	Dussault et al. (42)	4 cycles of 6 min; 425 MPa; 4°C and 37°C (*n* = 6)	Industrial-scale equipment (Model 135; Hiperbaric, Spain).	Diminution of initial milk bacterial flora	Preincubation of milk to 37°C seem to facilitate bacterial destruction by HPP treatment	No difference was observed in post- pasteurization bacterial loads between HPP and HoP treated milk (*p* > 0.16).
	Irazusta et al. (43)	Many conditions have been tested at 20°C: −400 MPa 5 min −450 MPa 5 min −500 MPa 5 min (*n* = 8)	S-IL-100-250-09 W (HP Food Processor, UK).	Microbiological Inactivation: Coliforms Total aerobic bacteria	Decreased bacterial concentration below detection limit Results for raw milk were not presented	NS (Median total counts obtained for HPP-treated samples at 400, 450, and 500 MPa were equal to median counts obtained for the HoP-treated samples)
	Pitino et al. (44)	500 MPa; 8 min T° not mentionned (*n* = 17)	Industrial-scale equipment (Model 135; Hiperbaric, Spain).	Diminution of initial milk bacterial flora	All methods of pasteurization increased the number of culture negative milk samples (<1 × 103 CFU/L) compared with pre pasteurization p (<0.05).	Higher proportion of samples with negative cultures after HPP than after Holder but NS (*p* = 0.06)
*B. cereus*	Jarzynka et al. (45)	450 MPa; 15 min; 21°C (*n* = 6)	Pilot-scale equipment (U 4000/65, Unipress Equipment, Poland).	Microbiological Inactivation: *E. coli* (1.2 × 10^7^ CFU/mL) *L. monocytogenes* (8.2 × 10^7^ CFU/mL) *S. aureus* (6.9 × 10^6^ CFU/mL) *C. sakazakii* (8 × 10^5^ CFU/mL) Sporulating *B. cereus* (4 × 10^5^ CFU/mL)	Complete inactivation	Total vegetative forms of the native microbiota were destroyed by HoP; no result for sporulating forms after HoP
Microbicidal activity	Barbarska et al. (46)	Many conditions have been tested: −450 MPa; 15 min −200 MPa; 10 min + 400 MPa; 10 min Inoculation of 6 × 10^7^ CFU/mL of *E coli*, post-HPP treatment; incubation for 2 h at 37°C. (*n* = 6)	Pilot-scale equipment (U 4000/65, Unipress Equipment, Poland).	Bactericidal activity against *E. coli*	NS (reduction of *E. coli* growth from 29.6 to 50.3%)	Not compared
	Kothari et al. (47)	1 cycle of 8 min; 500 MPa, 4°C Following HPP treatment, milk was spiked with CMV (~5 log PFU/mL) and incubated up to 4 h at room temperature. Post-incubation CMV titers were measured.(*n = 10*)	Industrial-scale equipment (Model 135; Hiperbaric, Spain).	Anti-cytomegalovirus activity	HPP-treated milk maintains anti-CMV activity comparable to raw milk. After a 30-min incubation period, 3.4 and 3.7 log PFU/mL were measured in raw and HPP-treated milk, respectively.	HoP-treated milk maintains anti-CMV activity comparable to HPP-treated milk after a 30-min post- pasteurization period. Not significantly different.
Viruses	Pitino et al. (48)	Many conditions have been tested at <10°C: −350 MPa 8 and 10 min −500 MPa 8 and 10 min −600 MPa 8 and 10 min	1 L-pilot unit Dustec Hochdrunktechnik GmbH (Germany).	Cytomegalovirus (CMV) Hepatitis A virus (HAV)	Reduction of CMV (initial load: 5.1 log PFU/mL) and HAV (initial load: 5.7 log PFU/mL) below the detection limit for all HPP treatments;	NS for reduction of CMV HoP was less effective than HPP treatments in reducing HAV in human milk (*p* < 0.05).

*p-value are indicated only when significant and/or reported in the original article; NS, not significantly different.

**Table 5:** Effects of HPP on quality of human milk: nutrients and bioactive factors retention.*

	Reference	HPP treatment parameters	Device	Milk components/properties	Impact of treatment (compared to raw milk)	Comparison HoP -HPP
Nutrients (Protein, Fat, Carbohydrates)	Marousez et al. (49)	4 cycles of 5 min; 350 MPa; 38°C Compression rate 1 MPa/s (*n* = 8)	Not precised	HMO Maillard reaction products: -Furosine -CML -CEL	NS NS NS NS	NS All components were statistically increased compared to HPP (*p* ≤ 0.01)
	Aceti et al. (28)	600 MPa; 3 min; 4°C (*n* = 5)	Industrial device AV-30 (Avure Technologies, Inc., OH).	Total amount of proteins	NS	Not compared
				Secretory IgA	Decreased (−39%) (significant reduction)	
				Lactoferrin content	Decreased (−24%) (*not significant*)	
	Zhang et al. (50)	400 MPa for 5 min, 20°C (*n* = 2)	Hippo 20 food isostatic press (Ilshin Autoclave, Daejeon, Korea)	casein proteins whey proteins MFGM (Milk Fat Globule Membrane) proteins	Relative abundance of some MFGM Proteins significantly altered Increased abundance of bile salt-dependent lipase and no change in the abundance of lipoprotein lipase in the MFGM fraction Lactoferrin unchanged (whey) Protein profiles less affected in the whey fraction, compared with the MFGM or casein fraction	MFG were aggregated by HoP. MFGM and casein proteins were affected by HoP Better preservation of the protein profile for HPP compared to HoP
	Wesolowska et al. (51)	Many conditions have been tested at ~20°C: −600 MPa 10 min −100 MPa 10 min + 600 MPa 10 min −200 MPa 10 min + 400 MPa 10 min −200 MPa 10 min + 600 MPa 10 min −450 MPa 15 min (*n* = 6)	pilot-scale equipment (U 4000/65, Unipress Equipment, Poland)	Lipase activity	A slight decrease was observed for all tested parameters. The decrease was less important for 200 + 400 MPa (17.8%) and for 450 MPa (−12.7%);	Better preservation in HPP-treated milk than HoP for these treatments (*p* < 0.05): −600 MPa 10 min −200 MPa 10 min + 400 MPa 10 min −450 MPa 15 min
				Free fatty acids (FAA) content	Decreased	Decreased (*p* < 0.05)
				Oxidative stability	Longer induction time (IT) for 600 MPa and 200 + 600 MPa (~ + 5%); decreased IT for other treatments (≥ − 3 min) (Longer IT is associated with better stability)	
				Fatty acid composition	Acids in milk after HoP at 450 MPa (*p* < 0.05). Significant decrease in the percentages of oleic acid and fatty	Decreased induction time (IT) (*p* < 0.05)
				Fatty acid distribution	Increase in polyunsaturated fatty acids and monounsaturated fatty acid and decrease in saturated fatty acid esterified at sn-2 position for all treatments except for fatty acid esterified at sn-2 position in milk treated at 200 + 600 MPa (NS)	NS between HoP and HPP below 450 MPa
				Carotenoids	Destructive effect on lutein+zeaxanthin; no change for β-carotene and a slight increase for lycopene	Destructive effect on lutein+zeaxanthin of HPP compared to HoP (*p* < 0.05)
	Pitino et al. (52)	500 MPa; 8 min (*n* = 8)	Industrial-scale equipment (Model 135; Hiperbaric, Spain).	Fatty acid composition Oxylipins (ng/ml)	NS Higher levels of some arachidonic-derived oxylipins following HPP can be a consequence of pressure-mediated quaternary structure stabilization of cyclo-oxygenase and lipoxygenase.	NS Lower concentration of a-linolenic acid oxylipin (*p* < 0.04) and higher concentration of EPA oxylipin (*p* < 0.05) in HoP-treated HM compared to HPP-treated HM
	Pitino et al. (44)	Many conditions have been tested at <10°C: −350 MPa 8 and 10 min −500 MPa 8 and 10 min −600 MPa 8 and 10 min	1 L-pilot unit Dustec Hochdrunktechnik GmbH (Germany).	Macronutrients content (g/L) Lactoferrin (g/L)	NS In absolute values, there was no statistical difference between raw milk and HPP treatments; once corrected for true protein, only the 600 MPa treatment of milk for 10 min shows a significant decrease in lactoferrin content compared to raw milk.	NS In absolute values, no statistical difference between HPP and HoP; once corrected for true protein, HoP shows a significant decrease in lactoferrin content compared to HPP treatments at 500 MPa or less (*p* < 0.05).
Bioactives factors	Zhang et al. (50)	400 MPa; 5 min; 25°C	Hippo 20 device (Ilshin Autoclave, Daejeon, Korea).	Lysozyme Xanthine oxidase Lactoperoxidase IgA Lactoferrin Lipoprotein lipase BSSL	NS NS NS NS NS NS NS	All statistically different from HPP (decreased) (*p* < 0.05)
	Mank et al. (23)	500 MPa; 5 min (*n* = 28)	pilot-scale equipment (Resato, Roden, Netherlands).	Insulin concentration (pmol/L)	NS	Not compared
	Jarzynka et al. (45)	450 MPa; 15 min; 21°C (*n* = 6)	pilot-scale equipment (U 4000/65, Unipress Equipment, Poland).	Lipase Leptin Adiponectin Insulin Hepatic growth factor	NS NS Decreased (−85%) NS Decreased (−21%)	Not compared
	Dussault et al. (42)	4 cycles of 6 min; 425 MPa; 4°C and 37°C (*n* = 6)	Industrial-scale equipment (Model 135; Hiperbaric)	IgA (μg/mL) IgG (μg/mL) IgM (μg/mL) Lysozyme (U/ml) Lactoferrin (mg/ml) Lipase (pg/mL) Cytokines (pg/mL)	NS NS NS for 4°C-HPP treatment NS NS for 37°C-HPP treatment NS NS	NS Significant decrease (p < 0.01) Significant decrease (p < 0.05) NS Significant decrease (*p* < 0.01) NS NS
	Koh et al. (53)	550 MPa; 5 min (*n* = 3)	Not precised	BSSL activity (U/mL)	Decreased (−38.4%)	Not compared
	Wemelle et al. (54)	4 cycles of 5 min; 350 MPa; 38°C Compression rate 1 MPa/s (*n* = 8)	Not precised	H_2_O_2_ (nM)	Significantly decreased	HPP treatment significantly decreased H_2_O_2_ level compared to HoP (*p* < 0.01)
				Vitamin A (mg/L)	NS	NS
				Vitamin E (mg/L)	NS	Not compared
				Total antioxidant capacity	NS	NS
	Wemelle et al. (55)	4 cycles of 5 min; 350 MPa; 38°C Compression rate 1 MPa/s (*n* = 8)	Not precised	Apelin (ng/ml) GLP-1 (ng/ml)	NS NS	Decreased in HoP (*p* < 0.05) Decreased in HoP (*p* < 0.0001)
	Irazusta et al. (43)	Many conditions have been tested at 20°C: −400 MPa 5 min −450 MPa 5 min −500 MPa 5 min (*n* = 8)	S-IL-100-250-09 W (HP Food Processor, UK).	IgA (μg/mL) IgM (μg/mL)	For statistical analysis, authors did not compare HPP with raw milk but with HoP-treated milk 86–101.5% of raw milk content 49–97.5% of raw milk content	Retention of IgA, IgM, and IgG in HM samples was at least 35.5, 23, and 22.5% higher in HPP-treated HM than in HoP-treated HM samples (*p* < 0.05)
				IgG (μg/mL)	98–104.5% of raw milk content	
				Lysozyme (×10^3^LU/mL)	125–128% of raw milk content	Significantly higher in HPP-treated HM (at least 52.5% higher) (*p* < 0.05)
				TGF-β2 (ng/mL)	69.9–88.5% of raw milk content	Significantly higher after the HPP treatment of more than 450 MPa (at least 15.6% higher) (*p* < 0.05)
				sCD14 (μg/mL)	28.3–111.2% of raw milk content	Significantly higher after HPP treatment (at least 28.2% higher) (*p* < 0.05)
				Nutritional components (lipids, proteins, lactose) (mg/mL) (energy) (Cal/mL)	Results for raw milk were not presented	NS
	Smyczynska et al. (56)	450 MPa; 15 min (*n* = 3)	Pilot-scale equipment (U 4000/65, Unipress Equipment, Poland).	microRNA content	Some miRNAs are degraded under high pressure. The diminution of miRNAs could decline functions of human milk.	Not compared
	Marousez et al. (57)	4 cycles of 5 min; 350 MPa; 38°C Compression rate 1 MPa/s (*n* = 8)	Not precised	Leptin	NS	Compared to HoP-treated HM, HPP treatment significantly increased concentration of insulin, nesfatin-1, cortisol, leptin, apelin and GLP-1 (*p* < 0.05) and reduced adiponectin by 52% (*p* < 0.01).
				Insulin (mU/L)	NS	
				Apelin (ng/ml)	Reduced (−20%)	
				Adiponectin (ng/ml)	Reduced (−50%)	
				Nesfatin-1	NS	
				Cortisol (ng/ml) GPL-1	NS Increased (+64%) → *maybe due to a release of GLP-1 from fat globules to the aqueous phase following HPP treatment*	
	Kontopodi et al. (24)	Many conditions have been tested: −400 MPa 5, 10, 30 min −500 MPa 1.5, 2 × 1.5, 3, 5 min −600 MPa 1.5, 2 × 1.5, 3, 5 min (*n* = 2)	Pilot-scale equipment (Resato, Roden, Netherlands).	Total protein concentration (mg/ml)	NS	Not compared
				Proteome	Most intense HPP treatment (600 MPa; 3 and 5 min) have a larger effect than other treatments	
				IgA	NS for 400 and 500 MPa treatments	
				Lactoferrin	NS	
				Lysozyme	NS	
				BSSL activity (%)	NS	
	Pitino et al. (44)	500 MPa; 8 min (*n* = 17)	Industrial-scale equipment (Model 135; Hiperbaric, Spain).	Macronutrients content (g/L)	NS (significant reduction in mean total carbohydrate content after HPP (*p* = 0.04))	Significant reduction in mean total carbohydrate content after HPP (*p* < 0.05).
				Folate (nmol/L)	NS	NS if compared to HPP
				Vitamin C (mg/L)	Decreased (−11 mg/L) (*76%*)	NS if compared to HPP
				BSSL (U/ml)	NS	Significant decrease (*p* < 0.05)
				Lactoferrin (g/L)	Decreased (−0.44 g/L)	Significant decrease (*p* < 0.05)
				Lysozyme (U/L)	NS	Lysozyme activity after HPP was higher (*p* < 0.05) than with HoP

*p-value are indicated only when significant and/or reported in the original article; NS, not significantly different.

**Table 6:** *In vivo*/*in vitro* metabolic studies on HPP effect.*

	Reference	HPP treatment parameters	Device	Milk components/properties	Impact of treatment (compared to raw milk)	Comparison HoP – HPP
*In vivo*/metabolic studies	Wemelle et al. (54)	4 cycles of 5 min; 350 MPa; 38°C Compression rate 1 MPa/s (*n* = 8)	Not precised	Level of antioxidant gene expression in the liver of mice treated with HPP-donor milk Inflammatory markers in liver and ileum of mice treated with HPP-DM	Increase of catalase and Superoxide dismutase mRNA expression → stimulation of antioxidant defense NS for other genes (Sod1, Gpx1, Gpx2, Nox1/2) Decrease in F4/80 mRNA expression (ileum) Decreased expression of Tnfɑ et F4/80 mRNA (liver) → reduced inflammation	Catalase and Sod2 mRNA expression were significantly decreased in HoP-treated HM (*p* < 0.05) After supplementation of mice with HPP-treated milk compared to HoP-treated milk: Decrease in F4/80 mRNA expression in both the ileum and (*p* < 0.05). Better stimulation of antioxidant defense and reduction of inflammation in both the ileum and liver
	Wemelle et al. (55)	4 cycles of 5 min; 350 MPa; 38°C Compression rate 1 MPa/s (*n* = 8)	Not precised	Glucose tolerance in mice Isotonic intestinal contraction in mice	Not compared	In mice supplemented with HPP-treated milk compared to HoP-treated milk: Improvement of glucose tolerance (*p* < 0.05) Decrease of the amplitude of duodenum contraction (*p* < 0.05)
*In vitro*	Zhang et al. (50)	400 MPa; 5 min; 25°C	Hippo 20 device (Ilshin Autoclave, Daejeon, Korea).	*In vitro* static digestion assays	HPP increased proportion of larger fat globule but HPP milk had a similar size distribution pattern as raw milk following a 60-min of gastric digestion. As in raw milk, lactoferrin remain largely undigested in HPP milk following gastric digestion.	Not compared

*p-value are indicated only when significant and/or reported in the original article.

NS, not significantly different.

#### 4.1.1 Effect on safety of HM

The last EMBA literature review (19) reported a destruction of *E. coli*, *S. aureus, L. monocytogenes,* and *Salmonella* sp. in HM within the 300–400 MPa pressure range.

Different HPP settings (400–600 MPa) have been tested to reduce bacterial load in DHM (24, 42–47) (Table 4). Total inactivation of *E. cloacae* and *S. aureus* (10^8^ CFU/mL) was obtained by subjecting milk to various pressure and holding time (400 MPa for 5, 10, and 30 min, 500 MPa for 1.5, 2 × 1.5, 3, and 5 min, 600 MPa for 1.5, 2 × 1.5, 3, and 5 min) (23). By applying a pressure of 450 MPa for 15 min, Jarzynka et al. (45) reported a total inactivation of *E. coli* (1.2 × 10^7^ CFU/mL), *L. monocytogenes* (8.2 × 10^7^ CFU/mL), *S. aureus* (6.9 × 10^6^ CFU/mL), and *C. sakazakii* (8 × 10^5^ CFU/mL). Rocha-Pimienta et al. (58) proposed a model to predict the inactivation of microorganisms by HPP of HM. By using this model, a reduction of 10^6^ CFU/mL of *S. aureus* and 10^7^ CFU/mL of *B. cereus*, in its vegetative form, was achieved by applying a pressure of 600 MPa for almost 4 min.

Sporulating bacteria such as *Bacillus cereus* are a major problem in human milk banks, and optimizing the HPP process to destroy such bacteria is still a challenge. Dussault et al. observed that the use of a higher temperature, 37°C rather than 4°C, during an HPP treatment of 4-cycles of 6 min each at 425 MPa, is more efficient in eliminating bacterial load naturally present in raw milk (42). When the treatment was performed at 37°C, the researchers observed an unequal destruction of the initial bacterial load (between 10^2^ and 10^3^ CFU/mL) according to the pools, with *B. cereus* surviving, probably due to bacterial spores resistant to HPP treatment (42). Applying a pressure of 600 MPa for almost 4 min, Rocha-Pimienta et al. also failed to destroy *Bacillus cereus* spores inoculated at an initial concentration of 10^7^ CFU/mL in milk (58).

Bactericidal and bacteriostatic activity of DHM is its ability to reduce the growth of milk pathogens through the presence of bioactive compounds, such as lactoperoxidase.

Barbarska et al. (46) processed milk samples in two ways: (1) 450 MPa for 15 min; or (2) 200 MPa for 10 min + 10 min break + 450 MPa for 10 min. They used a 0.2 mL inoculum with a concentration of 3 × 10^8^ CFU/mL of *E. coli* to inoculate 0.8 mL milk samples. After a 2 h-incubation at 37°C, the bactericidal capacity of HPP-treated milk against *E. coli* was preserved at 29.6% (450 MPa) and at 50.3% (200 MPa + 450 MPa). These results were not significantly different from those obtained with raw milk, for which a 46.6% reduction in *E. coli* growth was observed (*p* ≥ 0.34). These results suggest that, at these conditions, HPP treatment did not negatively affect the bactericidal activity of human milk against *E. coli*.

It should be noted that the elimination of contaminating pathogens by HPP has led to the elimination of all bacteria present in raw milk and likely to colonize infant gut in early life (59).

Pitino et al. (48) reported the reduction of CMV (initial load of 10^5^ PFU/mL) and hepatitis A virus (HAV) (initial load of 10^6^ PFU/mL) below the detection limit (4 PFU/mL) for multiple HPP treatment tested (350–600 MPa; 8–10 min). Furthermore, Kothari and colleagues (47) reported a maintained anti-CMV activity in milk treated by one HPP-cycle of 500 MPa for 8 min. Therefore, HPP used in these conditions preserves antiviral activity.

In summary, most pathogens currently present in HM, including 4 × 10^5^ CFU/mL of vegetative cells of *B. cereus*, and 10^5^–10^6^ PFU/mL of CMV and HAV are destroyed by HPP, and antimicrobial activity of HM is preserved (Table 4).

The HoP treatment shows a destruction of the milk bacterial load and the initial CMV load very similar to that of the HPP treatment (23, 41, 42, 46). HoP-treated milk maintains post–pasteurization anti-CMV activity comparable to HPP-treated milk (48). However, HoP is less efficient in destroying hepatitis A virus than HPP at a pressure equal or over 500 MPa (48).

#### 4.1.2 Effect on quality of HM

The 2019 review (19) indicates that an HPP treatment from 100 to 500 MPa, up to 10 min, allows retention of macronutrients when compared to raw milk. The new evidences on the effects of HPP on quality of HM are summarized on Table 5.

Macronutrients’ content is similar in raw milk and in HPP treated milk at 350, 500, or 600 MPa for 8 or 10 min (48). Moreover, in another study, the same research team detected no statistical difference in folate levels between raw milk and milk subjected to an HPP treatment at 500 MPa for 8 min, while vitamin C was destroyed by HPP treatment at 500 MPa for 5 min or more (44). In summary, HPP has no significant effect on nutrient content, except for vitamin C.

As emphasized in the past review (19), bioactive components are generally well preserved after HPP treatment with a few exceptions. Wesolowska and colleagues demonstrated that HPP treatment, especially at 450 MPa for 15 min, lead to almost complete retention of lipase activity (87.3%) (51). Other studies have also reported no loss of BSSL activity after HPP treatments of DHM at 400 MPa for 5 min (50), or 500 MPa for 8 min (46). However, lipase activity is decreased by HPP treatment above 500 MPa: a 62% retention of lipase activity in milk treated at 550 MPa for 5 min when compared to raw HM (*p* < 0.05) was reported (53). Wesolowska and colleagues (48) detected retention of only 16.5% of the lipase activity present in raw milk after a treatment at 600 MPa for 10 min.

Regarding protein, no change in lipoprotein lipase abundance in the milk fat globule membrane fraction was reported following HPP at 400 MPa for 5 min (60). In the same study, protein profiles were less affected in the whey fraction, compared to the milk fat globule membrane or casein fraction. Given these results from studies on HPP impact on lipase, this treatment has no or limited effect on lipase activity when used at pressure below 500 MPa.

Lysozyme is a major component of breastmilk and has been widely studied with respect to its retention in DHM after pasteurization processes and shown to be resistant to HPP treatment. By treating maternal milk at 400 MPa for 5 min, Zhang et al. (59) observed an increase of lysozyme activity to approximately 120% of the activity detected in raw milk. This result agrees with other studies, such as Pitino et al. (44) and Dussault et al. (42). Increase of lysozyme activity could be attributed to a partial unfolding of the lysozyme protein during the HPP treatment. This increases the surface area and lytic activity of the enzyme against the cell wall of *Micrococcus lysodeikticus* used for the turbimetric test. This results in an increased lysozyme activity (61).

Many discrepancies are observed for lactoferrin between the results obtained after different HPP protocols. Because of the high sensitivity of lactoferrin to HPP and to different heat treatments of milk, its measurement is often considered as a reference for evaluation of the impact of milk processing on its quality. From recent studies a trend has emerged: the higher the pressure, the lower the milk lactoferrin content. From 500 MPa for 8 or 10 min, Pitino et al. (48) observed a significant decrease in lactoferrin levels compared to raw milk. Lactoferrin analysis was performed by HPLC. At 600 MPa for 10 min, Kontopodi et al. suggest an aggregation of lactoferrin that could be associated to the protein aggregation observed by proteomics after treatment at 600 MPa for 3 or 5 min (24). Aceti et al. (28) (600 MPa, 3 min), Zang et al. (50) (400 MPa, 5 min), and Dussault et al. (42) (4 cycles of 425 MPa, 6 min) used ELISA to assess lactoferrin levels and all reported a non-significant decrease in lactoferrin after treatment. Kontopodi et al. also obtained non-significant decrease in lactoferrin after HPP treatment, proteins were analyzed by LC–MS/MS (24). However, the temperature of the treatment may have an impact on lactoferrin levels: Dussault et al. (42) observed a significant decrease (37%) when HPP is performed at lower temperature (at 4°C) compared to untreated milk.

Application of HPP at 400 MPa for 5 min did not affect IgA content of treated milk (50). Similarly, when applying 4-cycles at 425 MPa for 6 min each, there was no significant difference in IgA and IgG levels between raw milk and HPP-treated milk (42). Contrary to IgG and IgA, IgM is significantly reduced by a 4-cycles HPP treatment at 425 MPa and 37°C for 6 min (42). After a treatment of 5 min to 500 MPa, Irazusta et al. (43) reported a halved (−49%) IgM content when compared to raw milk.

Lactoperoxidase and xanthine oxidase are two natural enzymes found in breast milk that prevent the multiplication of bacteria. Zhang et al. (50) showed preservation of activity up to 110 and 90%, respectively, after HPP at 400 MPa for 5 min.

There are more than 160 different human milk oligosaccharides (HMOs), some of which exert beneficial effects on newborn development and protection (62, 63). Very little information on the impact of HPP on HMOs levels is known. Marousez et al. (49) quantified 22 major HMOs in raw milk and in milk sample that underwent 4 cycles of 5 min each of HPP treatment at 350 MPa. HMOs remained unaffected by this HPP treatment. The authors also studied the formation of Maillard reaction products (MRPs) as a result of chemical reactions between reduced sugars and amino groups in proteins. Marousez et al. (49) quantified 3 MRPs (furosine, N-epsilon-carboxymethyllysine and N-epsilon-carboxyethyllysine) in maternal milk exposed to the previously described HPP and concluded that this protocol avoids MRPs formation.

Of the 26 cytokines analyzed by Dussault et al. (42), only IL-8, IP-10, MCP-1, MIG, and TGF-β_2_ were detected at measurable levels, even in raw milk. No significant difference was observed between milk samples treated with 4 cycles of HPP treatment (425 MPa for 6 min) and untreated milk samples, suggesting that HPP do not affect these factors.

The sCD14 is a protein that detects bacteria by binding to lipopolysaccharides. While this protein is well preserved in milk after HPP treatment at 400 MPa for 5 min at 20°C (111% of the amount found in raw milk), increasing the pressure seems to have a deleterious effect on sCD14 since 51% and only 28% are retrieved after treatment at 450 MPa and 500 MPa, respectively (43). These results suggest that milk treated at more than 400 MPa may not provide preterm infants with an adequate response to inflammation in comparison to milk treated to low pressure.

Hormones are active factors present in human milk that contribute to the control of energy intake and gut maturation in newborns, both directly and indirectly. Thus, Marousez and colleagues (57) studied the impact of HPP treatment on 6 peptide hormones (leptin, insulin, adiponectin, apelin, GLP-1, and nesfastin-1) and a steroid hormone (cortisol). No significant loss of leptin, insulin, nesfastin-1, or cortisol was observed after 4 cycles of 5 min HPP treatment at 350 MPa compared with raw milk. Similarly, Jarzynka et al. (45) investigated hormone retention in human milk and reported comparable levels of leptin and insulin in milk samples treated with HPP at 450 MPa for 15 min and in untreated samples. Preservation of insulin levels is also reported by Mank et al. (23) following a different HPP protocol (500 MPa for 5 min). However, in a few studies, HPP of human milk seems to have a deleterious effect on the retention of some hormones present in milk since both Marousez et al. (57) and Jarzynka et al. (45) observed a reduction of adiponectin by 50 and 85%, respectively, after a treatment of 4 cycles of 5 min at 350 MPa or at 450 MPa for 15 min (holding time). Marousez et al. (57) also reported a significant decrease in apelin level (20%) after HPP treatment.

HPP can affect the quality of HM with respect to certain nutrients and bioactive factors. For example, oxylipin which derived from PUFAs by oxidation and may be involved in resolving inflammation (52), have been shown to be destroyed by HPP treatment at 500 MPa for 5 min or more. For lutein, which has been shown to have antioxidant and anti-inflammatory properties, Wesolowska and colleagues (51) detected a retention of only 39.8% of lutein present in raw milk, after a treatment at 600 MPa for 10 min. Some miRNAs are also degraded under high pressure, mainly miRNAs related to pathways involved in extracellular matrix -receptor interactions, cell proliferation and metabolism regulation (56). Below 500 MPa, HPP treatment has limited effect on the quality of human milk except for vitamin C, IgM, and apelin.

Compared to HoP, HPP has no effect on the macronutrient composition of milk (48) except for a significant decrease in total carbohydrate content in HPP-treated HM (41). Zhang and colleagues, however, showed that MFG tended to aggregate in HoP-treated milk and that milk fat globule membrane proteins as well as casein were affected (50). Polyunsaturated fatty acids and monounsaturated fatty acids were increased in milk after HPP at 450 MPa (*p* < 0.05), while saturated fatty acids decreased (51). Many authors have shown significant decrease in bioactive factors in milk following HoP compared with HPP. Thus, immunoglobulins (42, 43, 50), lactoferrin (42, 44, 48, 50), lysozyme (43, 44, 50), lipase activity (24, 44, 45, 50, 51, 53) and many hormones (54, 57) are largely affected by HoP.

#### 4.1.3 Effect demonstrated *in vivo* and *in vitro* models

A growing number of experiments are being conducted to investigate the effect of HPP-treated HM on metabolism and physiology (Table 6). An increase in the expression of antioxidant genes (catalase and super dismutase) and in glucose tolerance was observed in mice fed with HPP-donor milk, as demonstrated by Wemelle et al. (55). Authors also showed a decrease in inflammation markers (F4/80, TNF-α) in these animals. After a 60-min *in vitro* gastric digestion assay, Zhang et al. (50) reported a particle size distribution of HPP-milk similar to that of digested raw milk despite the initial observation of an increased proportion of larger fat globules in HPP-treated milk (400 MPa for 5 min) compared with raw milk. Possible aggregation of MFG may occur during HPP treatment since Ye et al. (64) also observed that α-lactalbumin, κ-casein, as well as β-lactoglobulin, associate with milk fat globule membrane proteins through disulfide bonds after HPP treatment.

Importantly, as in raw milk, Zhang and colleagues observed that lactoferrin in HPP-treated milk (400 MPa, 5 min) remained largely undigested after the digestion test (50). Given the antimicrobial properties of lactoferrin, this resistance to digestion is important for regulating the gut microflora of preterm infants.

Studies comparing mice fed HoP- and HPP-treated milk showed a loss of antioxidant defenses and an increase of inflammatory markers in animals fed HoP-treated milk (54).

### 4.2 HPP discussion

The lack of consensus regarding a “gold standard” HPP protocol makes that each research team develops its own protocol, making it very complex to navigate through the different results. Moreover, all technical details and HPP parameters are not always efficiently mentioned by authors, thus compromising repeatability of an HPP protocol. In order to completely destroy bacterial spores, several parameters must be considered and monitored during HPP treatment of milk: pressure level, temperature, compression and decompression rates, number of cycles and duration of each cycle. All these parameters should be presented by authors. Studies reported bacterial spores resisting to HPP treatment using pressure higher than 400 MPa (42, 58, 65). This phenomenon was studied in more detail by Wuytack et al. using *B. subtilis* spores. This group observed incomplete spore germination after one 600-MPa treatment. At this pressure, the small acid-soluble spore proteins (SASPs) would not be degraded, thus interrupting the germination process (66). In contrast, when pressure between 50 and 300 MPa is applied at 40°C for about 30 min to sporulated bacteria, germination is activated by the stimulation of nutrient receptor, leading the bacteria to act as if the environment is no longer hostile and to germinate into vegetative cells, which would then be destroyed by subsequent HPP cycles (67) underlying the importance of the number of cycles. It is also crucial to present the compression time leading to a progressive increase in pressure, allowing bacterial spores to germinate and bacteria to return to their vegetative form as previously demonstrated for another *Bacillus* sp. (68). Lastly, the initial milk temperature is also important since treatment above 30°C seems to facilitate the germination of bacterial spores which are eliminated by subsequent treatments (69). Dussault et al. (42) have shown that a pressure of 425 MPa tend to be more effective in destructing bacterial spores when milk samples are previously heated and treated at 37°C, than when they are treated at 4°C. This synergy between pressure, number of cycles and temperature is also observed and well described by Van Opstal and colleagues (69). Authors demonstrated that germination of bacterial spores (6 log) is efficient when milk temperature is above 30°C even at low pressure (200 MPa for 30 min). The bacteria are then eliminated by subsequent treatment. All these parameters have to be considered when establishing an HPP protocol in order to ensure safety and quality of DHM. The first review of the EMBA working group (19) reported the pioneering work of Demazeau et al. (70) based on an original protocol for using HPP: 4 cycles at 350 MPa, during a 5-min holding time, at a temperature of 38°C, with a compression/decompression rate of 1 MPa/s and a latency time of 5 min (at normal pressure) between each cycle. In these specific conditions, HPP treatment was able (i) to destroy at least 10^6^ CFU/mL bacteria, which meets the definition of a sterilization/ pasteurization process (ISO14937) (71), including multidrug-resistant *S. aureus*, and (ii) preserve lipase, lactoferrin, and lysozyme (70). These results still need to be verified. Only a comparison between such a multi-cycle process and such a single-cycle treatment, for the destruction of *B. cereus and B. subtilis* spores at a similar level, optimally at 10^6^ CFU/mL, would allow to conclude on the effectiveness of the treatment.

At present, to our knowledge, no HMB uses HPP as a pasteurization method. All the devices on the market are used for research and development purposes only. Clinical studies demonstrating all the benefits of using HPP rather than HoP are still needed before the widespread implementation of HPP into milk bank operations. Research so far, however, has been able to show that HPP would ensure comparable or even improved safety of DHM compared to HoP, in addition to allowing better preservation of nutrients and bioactive components (24, 42, 43, 45, 48, 50, 53, 55). As previously shown, preterms’ growth rate during their hospital stay depends on overall quality of lipidome (72). Better preservation of human milk lipids would therefore be an advantageous asset for human milk banks. As the cost of the device is high, a full medico-economic evaluation is needed before its routine use in HMBs (73).

## 5 Other new advanced technologies of HM treatment

A literature review has been conducted by searching separately the following terms in the Title/Abstract/Keywords domains in Scopus, and in Title/Abstract/MeSH terms in PubMed, limiting the search in the time range 2019–2022: (“human milk” OR “donor milk” OR “breastmilk”) AND search n°1 (“Ultraviolet C radiation” OR “UV-C”); search n°2 (“Microwave Treatment”); search n°5 (“Gamma-Irradiation”); search n°6 (“Thermoultrasonication” OR “TUS”); search n°7 (“Freeze-drying” OR “FD” OR “lyophilization”).

### 5.1 Ultraviolet C (UV-C) radiation

Short-wavelength ultraviolet C (UV-C) radiation is a treatment commonly used in the food industry (range of waves: 200–280 nm). Despite penetrating the microorganism, ultraviolet light has no effect on the organoleptic characteristics of the food being treated. It works by damaging the nucleic acids in these organisms and prevent them from carrying out essential physiological processes. However, depending on the product’s optical characteristics, UV light can only penetrate food materials a few millimeters. The liquid’s turbidity and/or color affect the optical absorption coefficient. Milk and other cloudy foods cannot be penetrated by UV radiation. This poses a problem when treating routinely significant quantities of donor HM in HMBs making necessary to expose the milk to the UV-C in a thin layer.

Since our last revision, some papers have increased the knowledge on the effects of UV-C on HM. Thus, Kontopodi et al. (24) demonstrated that UV-C may be considered as a suitable alternative to HoP, since it was able to ensure sufficient microbiological inactivation while at the same time better preserving the bioactive components of DHM. The milk was treated in a sterile beaker glass filled with 140 mL of milk with a UV-C lamp placed diagonally, while magnetically stirred. A UV-C dosage of 4,863 J/L was the UV-C dosage effective in causing a >5 log reduction of *E. cloacae*, *E. coli* and *S. epidermidis* counts. This dosage did not cause a significant reduction on the levels of IgA, lactoferrin, lysozyme or BSSL, and the changes in the DHM proteome after UV-C treatment were minimal. By subjecting DHM to the same treatment, Mank et al. (23) showed that UV-C treatment preserves insulin in DHM. In addition, Almutawif et al. (74) showed that treatment with UV-C at a dosage of 2,259 J/L reduces *S. aureus* growth with similar kinetics to raw milk, thus confirming that the immune protein bioactivity present in HM is not affected by the treatment with UV-C irradiation. Moreover, in parallel with this reduced growth, no enterotoxin production was detected in UV-C treated DHM. Also in this case, the DHM was treated in a glass beaker with a germicidal UV-C lamp introduced diagonally.

The absence of a proper equipment in the setting of a HMB presents the biggest obstacle to evaluating this technology. However, some promising equipment have been analyzed in the treatment of other products than human milk, like donkey milk or swine plasma. This equipment, a pilot-scale SurePure Turbulator UV-C reactor system, creates a turbulent flow and is designed for continuous flow inactivation of turbid fluids. Considering that it is operating at a flow rate of 4,000 L/h, depending on the UV dose applied, the time to treat 1 L would be around 1 min. Papademas et al. (75) tested this device with 7 L of artificially inoculated (*L. innocua*, *S. aureus*, *B. cereus*, *Cronobacter sakazakii*, *E. coli*, *Salmonella enteriditis*; 1 × 10^5^ CFU/mL) donkey milk and observed a complete inactivation at doses in the range of 200–600 J/L, except for *L. innocua* who need a dose of 1,100 J/L. By using the same prototype, a series of papers (76, 77) of the same group confirmed the capability of Sure Pure Turbulator UV-C treatment system to inactivate significant levels of swine enveloped and non-enveloped viruses as well as bacterial strains (*Salmonella typhimurium*, *Salmonella choleraesuis*, *Enterococcus faecium*) inoculated in commercially collected porcine or bovine plasma.

Regarding the effect of UV-C on the elimination of bacterial spores present in human milk, there are no published data. However, there are promising data on the ability of UVC irradiation to eliminate bacterial spores present in different liquids such as orange juice (78), distilled water (79), coconut water (80), platelet concentrates containing Ringer’s solution (81) or grape and apple juices (82).

### 5.2 Microwave treatment

Almost all international procedural guidelines forbid using household microwaves to defrost or heat-treat HM. This advice is mainly based on two studies that showed how using a household microwave to treat HM caused degradation of IgA and lysozyme and a quicker development of *E. coli* (83, 84). However, the methodology employed in these articles have been called into doubt (85) and a growing number of papers demonstrate that this technique is more harmless than previously believed (86–93).

To treat samples of DHM by microwave heating (MWH) and examine the impact on bioactive components of human milk, Malinowska-Panczyk et al. (93) evaluated a prototype microwave device where the temperature is maintained constant for a short period of time. While baseline lipase and TGF-B2 levels were lowered, like in milk that had been treated with HoP, lactoferrin and IgA were better conserved. Additionally, the treatment with MWH had a greater detrimental impact on the concentration and activity of lysozyme. Inoculated microbiota (1 × 10^5^ CFU/mL) was also shown to be inactivated following microwave heating at 62.5°C or 66°C for 5 or 3 min, respectively. The same team reported that, compared to the HoP method, pasteurization of HM using MW heating under controlled circumstances resulted in decreased loss of Brain derived neurotrophic factor and Glial cell line-derived neurotrophic factor (91).

Two additional studies demonstrated that samples microwave-treated at 62.5°C for 5 min had enzyme activities that were comparable to or greater than those subjected to HoP (88), and that the concentration of macronutrients, fatty acids, lipid peroxides, and α-lactalbumin after using MWH at 62.5°C for 5 min was on a par with that of raw milk, and furosine was not formed. Malonaldehyde and protein carbonyls (PC) levels in DHM slightly increased after MWH treatment. Malonaldehyde concentration in DHM was, however, lower due to MWH than it was following the use of convection heating. Mikawa et al. (92) reported that human CMV was completely destroyed from HM by MWH treatment at 500 W for 40 s. After a treatment at 2450 MHz with a maximum power of 300 W, at 60°C for 30 s, Leite et al. (86) detected the same decrease of microbial contamination in experimentally inoculated human milk (*S. aureus* and *S. typhimurium*, 10^6^ CFU/mL) as HoP. The same group reported in a different study (87), that immunoglobulins and lactoferrin did not significantly decrease in concentration following microwave treatment (60°C for 30 s, 65°C for 15 s), and oligosaccharides and fatty acids were not considerably altered. IgA, IgG, IgM, and lactoferrin, on the other hand, fall by 47, 45, 68, and 85%, respectively, following HoP.

### 5.3 Combination of freeze-drying (FD) or lyophilization and gamma irradiation

Gamma-irradiation is another non-thermal treatment that has been tested. Blackshaw et al. (94, 95) used 2 kGy gamma irradiation to treat freeze-dried DHM samples (moisture 2.2%), and they found no significant change in the protein profile or end-products of lipid oxidation. Additionally, this method lowered the amount of bacteria inoculant like HoP, without decreasing the DHM’s antibacterial effectiveness. There is no published data on the effect of this technique on the elimination of bacterial spores present in human milk. However, gamma irradiation has destructive capacity on the bacterial spores present in apple and orange juices (96) and deionized water (97).

Freeze-drying (FD), when utilized in HMBs, may have advantages for room temperature storage and transportation. FD is a low temperature dehydration process that involves freezing the product and lowering pressure, removing the ice by sublimation, contrary to conventional dehydration methods that evaporate water using heat (98). Sublimation allows the passage from the solid state to the gaseous state without passing through the liquid state. Freeze-dryer should be a high-quality device compatible with aseptic clean room operations allowing the production of sterile products. FD is the technique of choice for drying high-value products as the low operating temperatures allow preservation of product quality. Freeze-dried products can be easily re-hydrated. FD enables in-packaging pasteurization of dried DHM powder with a longer shelf-life, stored at ambient temperature, and easier to transport, which reduces storage and transportation costs. It can help to increase availability for newborn babies in hospitals or other places in demand, such as in crisis conditions for humanitarian aids. Main limitation for FD use is the very high cost of high-quality freeze-dryer. It requires high technical expertise and freeze-dryers usable routinely in HMBs. Therefore, it can be used only for large amounts of high-value products. Freeze-drying should not be confused with spray-drying, which is a process used to produce powdered formula or fortifiers, but uses very high temperatures, and have more deleterious effects than FD on milk components (87). Effects of freeze-drying on HM properties were reported in 8 studies between 1983 and 2020, only 3 after 2019. Most studies were performed using a laboratory bench freeze-dryer that can only process a limited volume of milk and requires prolonged (72 h) exposure to pressure, both being not compatible with the requirement of routine HMB operation (99–101). Milk properties of freeze-dried were preserved, even after few months of storage. Oliveira et al. demonstrated preservation of osmolality and stability of nutritional content in freeze-dried human milk samples that were subsequently stored for 3 and 6 months (99). Lactoferrin, lysozyme, total anti-antioxidant capacity and fat remained stable regardless of time (6 weeks) and temperature (5°C and 25°C) of storage (100). Only the activity of superoxide dismutase was significantly reduced in the stored freeze-dried samples, mainly during the first week of storage. However, that decrease was significantly less than the decrease reported during freezer storage of liquid HM (100).

As freeze-drying has no effects on microorganisms, it needs to be associated with a process ensuring microbiological safety (102). Recently, techniques have been tested in association with FD. Combination of high positive pressure (HPP) and FD preserved better the nutritional value than HoP and offered microbiological safety of DHM (45). Combination of FD and then low-dose (2 kGy) gamma irradiation shows no post-treatment difference in lipid oxidation end-products and variation in protein profile (94). This hybrid method also preserves antimicrobial properties and enables bulk pasteurization within sealed packaging of powdered DHM (93).

### 5.4 Thermoultrasonication (TUS)

Thermoultrasonication (TUS) is a process that combines ultrasonication (microbubble formation and their rapid collapse though inertial cavitation) with mild heating. By using a laboratory configuration of a sonifier and a circulating bath, Mank et al. (23) described that the treatment of DHM (60 W, 6 min: 1080 kJ/L) did not affected the concentration of insulin.

By testing two different conditions, 40 W, 9 min (1,081 kJ/L) and 60 W, 9 min (1,620 kJ/L), Kontopodi et al. (24) observed that all tested conditions achieved a >5-log10 reduction for all bacterial strains analyzed. In addition, the retention of sIgA, lysozyme and lactoferrin showed a decreasing tendency with increasing TUS intensity and exposure time, whereas BSSL retention decreased significantly, that is a pattern of protein damage similar to that of HoP-treated DHM. By using LC–MS/MS-based proteomics analysis, the authors described a pattern of protein damage like the one observed after HoP treatment of DHM.

Regarding the effect of TUS on the elimination of bacterial spores present in human milk, there are no published data. In addition, TUS have also demonstrated their harmful effect on bacterial spores present in water or cow’s milk (103).

## 6 Discussion and conclusion

This paper presents the new most advanced technologies of HM treatment realizing a literature search from 2019 to the end of 2022 as a continuation to the first EMBA paper on processing of DHM in 2019. In this sense, two of the technologies described in our previous paper, HTST pasteurization and HPP, are available on the market today. The only technique among those described in this paper that is regularly used in some HMB is HTST. A first clinical trial to compare the incidence of microbiological proven late onset sepsis, NEC, ROP, mortality, oxygen supplementation, and infant growth in newborns <1,000 g that in the first 28 days of life need to be supplemented with donor milk pasteurized by HTST method versus the Holder method has been recently completed (NCT04424667) in two hospitals, the Neonatology Services of the Hospital 12 de Octubre and the Hospital de La Paz in Madrid, Spain, and the results will be available soon. Recently, a randomized clinical trial funded by Polish Medical Agency Research has just begun, on the prevention of cow’s milk allergy through DHM supplementation. In this trial one arm of the intervention is HPP treated HM (Wesolowska, personal communication).

Recent evidence confirms that HTST pasteurization of HM allows a higher retention of bioactive factors, such as immunoglobulins, including sIgA, and lipase, when compared to HoP. It has also been observed that HTST processing resulted in improved preservation of the nutritional quality of HM than HoP, since relevant thermosensitive components, such as phospholipids, PUFAs and BSSL, were less affected. At present no other technique has been implemented in HMB practice, demonstrating a Technology Readiness Level of TRL9, and clinically demonstrated as alternative to HoP in a clinical trial. Concerning HPP, research so far has been able to show that this methodology would ensure comparable or even improved safety of human milk compared to HoP: most bacteria currently present in human milk, including the vegetative forms of *Bacillus cereus*, are destroyed by HPP (provided the results will be confirmed in further studies), and antimicrobial activity of HM is preserved. In addition, HPP allows better preservation of nutrients and bioactive components in comparison to HoP (24, 42, 43, 48, 50, 53, 55). However, the fact that several methodological approaches are used makes extremely difficult to compare studies. There is no doubt that determining the winning conditions for HPP should be a focus for future research. However, HPP as well as HTST seem to be excellent alternatives to HoP for ensuring safety, and quality of human milk for preterm babies is quite comparable to that of raw milk. Regarding other technologies for processing of HM, particular attention should be paid to the determination of parameters to avoid the loss of major components and/or biological activities while ensuring the destruction of both pathogenic bacteria and viruses. On the other hand, FD has no major effects on milk properties and offers advantages such as extended shelf-life, reduced storage and transport costs, larger capacity for storage at room temperature. It has to be associated with a process enabling microbiology safety of milk, such as HoP, HPP or Gamma irradiation. The main limitations are the costs and the required technical expertise to produce a safely dehydrated DHM.

The aim of this study is to evaluate what is new in the processing of DHM from a literature search of the period 2019–2022, and to compare the results derived from the new technologies with the results derived from traditional HoP. It is not our intention to make a classification of the new technologies based on the results obtained, because there are very few studies comparing the new methodologies and the results are scarce and not significant. The effects of the new technologies on safety and quality of human milk have been presented and discussed in the previous sections of this paper. Another thing that may be interesting for the reader is to evaluate the feasibility of the new technologies within HMBs with a practical approach, considering financial, human and technical aspects.

Regarding HTST, there are at present two devices available on the market (SIVE Fluid Systems, Alcalá de Henares, Madrid, Spain, and Labor Baby, Tribiano, Milan, Italy). They can be easily allocated in a HMB and both can be utilized by bank staff after a training course. The cost is higher than the cost of a traditional pasteurizer, and it can be addressed by many banks in high-income countries but not in low- and medium-income countries. HPP is a more sophisticated technology that seems able to inactivate *B. cereus* spores (even if this aspect has to be evaluated more deeply). The utilization of the device needs a high specialized and well-trained staff, a situation not easy to realize in the majority of HMBs. The dimensions of the instrument, and its allocation in HMBs is not compatible with the space available in the vast majority of HMBs. The cost of DHM processed with HPP is estimated to be 7 times higher than that of the milk treated with HoP. Even if we consider that the inactivation of *B. cereus* could reduce the amount of DHM discarded, it is very unlikely that the saving of money deriving from a better utilization of DHM is able to compensate the very high cost of the processing. Future studies should be carried out to assess the actual cost/effectiveness of HPP methodology. At present, no HMB uses HPP as a pasteurization method. All the devices on the market are used for research and development, waiting for a miniaturization of the machinery in order to be easily placed and routinely utilized for human milk processing in HMBs. FD is an emerging technology that is becoming more and more important in this period due to the possibility to increase the utilization of human milk in emergencies (war, epidemics, and natural disasters). Main limitations for FD use is the very high cost of high-quality freeze-dryer. UV-C radiation, TUS, microwave treatment and gamma-irradiation are all promising technologies but at the moment they are under evaluation in a lab phase and many practical problems have still to be solved before their utilization in a human milk bank environment.

HM processed by HMBs is destined to very vulnerable babies. Since HM may contain potential pathogenic microorganisms and viruses for these babies, their effective removal by pasteurization is a major concern for HMBs. A predominance of *Staphylococcus* species has been reported by several studies, but other miscellaneous bacteria have been identified in HM samples, including *Klebsiella* sp., *Streptococcus* sp., *Pseudomonas* sp., and *Enterobacter* sp. (104). Contamination of breastmilk may occur from a variety of sources including skin, breast pump and environment, and cases of severe sepsis caused by these microorganisms, transmitted by unpasteurised HM to premature babies, have been reported (105–108). *Bacillus cereus*, an environmental organism, can cause fatal infections in vulnerable patients such as premature babies. *B. cereus*, in its sporulating form, can survive in adverse conditions, making it very difficult to eliminate, even by pasteurization. *B. cereus* is an opportunistic microorganism that can cause nosocomial infections, including sepsis and digestive infections in immunocompromised newborns. Moreover *B. cereus* produces toxins, that may intoxicate infants even in the absence of vegetative forms and are heat resistant (109). While unprocessed HM has been pointed out as a potential source of *B. cereus* (110), no causal link between ingestion of banked milk and *B. cereus* infection in preterm babies has been confirmed according to Lewin et al. (111). Contamination could rather be attributed to immediate environment contamination, such as ventilation or invasive care equipment. Nonetheless, several publications show great interest in the presence of *B. cereus* in HM, whether it is in its vegetative or sporulating form.

Development of any sterilization/pasteurization process should not allow the growth of any pathogenic bacteria in treated milk and ensure the destruction of at least 10^6^ CFU/mL of reference bacteria and viruses (70). This should be considered when evaluating a new technology and developing an HM treatment protocol. Moreover, the establishment of an effective HM processing method in an HMB should ensure that maximum components and biological activity are retained in processed milk compared to raw milk. Consensus about the best techniques for analyzing these components will allow for consistency in results, considering that different factors may influence the results: i.e., the detection method (ELISA kit, HPLC, mass spectrometry) and the value assessed (concentration, activity). The consequences of partial elimination of pathogens and/or a loss of essential nutrients and bioactive components in milk for vulnerable premature babies could be devastating. These newborns need to grow rapidly and to develop their digestive and immune systems effectively, among other things, to ensure their survival. As previously mentioned, infection can cause fatal sepsis and severe injury, while a lack of essential components can compromise proper development. Moreover, it is also important to keep in mind the programming effect of a deleterious perinatal nutrition on later life metabolic and intestinal heath. For these reasons, it is relevant to assess the health benefits of donor’s milk after treatment, which are currently being evaluated *in vitro* and through animal studies, but not actually analyzed in a human population. Milk banks must be aware of these issues when choosing a method of human milk processing.

## Author contributions

GEM: Writing – original draft, Writing – review & editing. MeG: Writing – original draft, Writing – review & editing. CP: Writing – original draft, Writing – review & editing. NG: Writing – original draft, Writing – review & editing. DE-V: Writing – original draft, Writing – review & editing. KK: Writing – original draft, Writing – review & editing. TC: Writing – original draft, Writing – review & editing. EB: Writing – original draft, Writing – review & editing. C-YB: Writing – original draft, Writing – review & editing. RB: Writing – original draft, Writing – review & editing. JC: Writing – original draft, Writing – review & editing. AG: Writing – original draft, Writing – review & editing. CG: Writing – original draft, Writing – review & editing. DLa: Writing – original draft, Writing – review & editing. DLe: Writing – original draft, Writing – review & editing. J-CP: Writing – original draft, Writing – review & editing. AW: Writing – original draft, Writing – review & editing. SA: Writing – original draft, Writing – review & editing. LC: Writing – original draft, Writing – review & editing. MaG: Writing – original draft, Writing – review & editing.

## In memoriam

This paper is dedicated to Iolanda Minoli, who passed away during the preparation of the manuscript.

## References

[1] Cassidy TM. A world history of milk banking: 100 years in 20 minutes. [Invited plenary]. Vienna, Austria: Centenary Celebration of the Donor Human Milk Banking (2009).

[2] Moro GE. History of milk banking: from origin to present time. Breastfeed Med. (2018) 13:16–7. doi: 10.1089/bfm.2018.29077.gem 29624424

[3] Cassidy TM. Historical research: more than milk: the origins of human milk banking social relations. J Hum Lact. (2022) 38:344–50. doi: 10.1177/08903344221082674, PMID: 35352603 PMC9016667

[4] Cassidy T Dykes F. Banking on milk: an ethnography of donor human milk relations. London: Routledge (2019).

[5] Budde C. C.L.G. (1908). US Patent No. 779637.

[6] Anonymous. “‘Buddeized’ Milk”. Sci Am. (1903) 88:413–3.

[7] Glickstein M.. A study of iodization and buddeization on the enzymes, bacterial content, physical and chemical properties, and nutritional value of milk. Masters of Science Thesis, University of Massachusetts Amherst. (1937). Available at: https://scholarworks.umass.edu/theses/1556

[8] Soxhlet F. Ein verbessertes Verfahren der Milch-sterilisierung. Miinchener Medicinishe Wochenschrift. (1891) 38:335–8.

[9] Westhoff DC. Heating milk for microbial destruction: a historical outline and update. J Food Prot. (1978) 41:122–30. doi: 10.4315/0362-028X-41.2.122, PMID: 30795181

[10] Rankin SA Bradley RL Miller G Mildenhall KB. A 100-year review: a century of dairy processing advancements—pasteurization, cleaning and sanitation, and sanitary equipment design. J Dairy Sci. (2017) 100:9903–15. doi: 10.3168/jds.2017-13187 29153179

[11] Wolf JH. Saving babies and mothers: pioneering efforts to decrease infant and maternal mortality In: Ward JW Warren C, editors. Silent victories: The history and practice of public health in twentieth century America. Oxford: Oxford University Press (2006).

[12] Cohen M Ryan H. From human dairies to milk riders: a visual history of milk banking in New York City, 1918-2018. Front J Women Stud. (2019) 40:139–70. doi: 10.5250/fronjwomestud.40.3.0139

[13] Balci AT Wilbey RA. High pressure processing of milk-the first 100 years in the development of a new technology. Int J Dairy Technol. (1999) 52:149–55. doi: 10.1111/j.1471-0307.1999.tb02858.x

[14] Hite BH. The effect of high pressure preservation of milk. West Virginia Agric Exp Station Bull. (1899) 58:15–35.

[15] Franch A Audí C Ramírez-Santana C Permanyer M Pérez-Cano FJ Moltó-Puigmartí C. Banked human milk treatment and immunoactive factors content. Comparison with high pressure processing. Proc Nutr Soc. (2010) 69:E288. doi: 10.1017/S0029665110000777

[16] Christen L Lai CT Hartmann B Hartmann PE Geddes DT. Ultraviolet-C irradiation: a novel pasteurization method for donor human milk. PLoS One. (2013) 8:e68120. doi: 10.1371/journal.pone.0068120, PMID: 23840820 PMC3694044

[17] Swanson KW. Human milk as technology and technologies of human milk: medical imaginings in the early twentieth-century United States. Women's Stud Q. (2009) 37:20–37. doi: 10.1353/wsq.0.0160

[18] Banking on the Body. The market in blood, milk, and sperm in modern America. Cambridge: Harvard University Press (2014).

[19] Moro GE Billeaud C Rachel B Calvo J Cavallarin L Christen L. Processing of donor human milk: update and recommendations from the European milk Bank Association (EMBA). Front Pediatr. (2019) 7:1–10. doi: 10.3389/fped.2019.00049 30873395 PMC6403467

[20] Hamprecht K Maschmann J Müller D Dietz K Besenthal I Goelz. Cytomegalovirus (CMV) inactivation in breast milk: reassessment of pasteurization and freeze- thawing. Pediatr Res. (2004) 56:529–35. doi: 10.1203/01.PDR.0000139483.35087.BE, PMID: 15295084

[21] Terpstra FG Rechtman DJ Lee ML Hoeij K Van Berg H Engelenberg FAC. Antimicrobial and antiviral effect of high-temperature short-time (HTST) pasteurization applied to human milk. Breastfeed Med. (2007) 2:27–33. doi: 10.1089/bfm.2006.0015 17661617

[22] Hu Y Kontopodi E Mank E van den Akker CHP van Goudoever JB Hettinga K. Processing methods of donor human milk evaluated by a blood plasma clotting assay. Innov Food Sci Emerg Technol. (2022) 76:102938. doi: 10.1016/j.ifset.2022.102938

[23] Mank E Kontopodi E Heijboer AC van Elburg RM Hettinga K van Goudoever JB. Thermoultrasonication, ultraviolet-C irradiation, and high-pressure processing: novel techniques to preserve insulin in donor human milk. Clin Nutr. (2021) 40:5655–8. doi: 10.1016/j.clnu.2021.09.028, PMID: 34666256

[24] Kontopodi E Boeren S Stahl B van Goudoever JB van Elburg RM Hettinga K. High-temperature short-time preserves human milk’s bioactive proteins and their function better than pasteurization techniques with long processing times. Front Pediatr. (2022) 9:798609. doi: 10.3389/fped.2021.798609, PMID: 35127595 PMC8811466

[25] Klotz D Jansen S Gebauer C Fuchs H. Handling of breast milk by neonatal units: large differences in current practices and beliefs. Front Pediatr. (2018) 6:235. doi: 10.3389/fped.2018.00235, PMID: 30234076 PMC6131667

[26] Manzardo OA Toll LJ Müller K Nickel E Jonas D Baumgartner J. Novel heat treatment protocol for human milk. Front Pediatr. (2022) 10:990871. doi: 10.3389/fped.2022.990871, PMID: 36330365 PMC9623327

[27] Capriati T Goffredo BM Argentieri M De Vivo L Bernaschi P Cairoli S. A modified holder pasteurization method for donor human milk: preliminary data. Nutrients. (2019) 11:1139. doi: 10.3390/nu11051139, PMID: 31121859 PMC6566761

[28] Aceti A Cavallarin L Martini S Giribaldi M Vitali F Ambretti S. Effect of alternative pasteurization techniques on human milk’s bioactive proteins. J Pediatr Gastroenterol Nutr. (2020) 70:508–12. doi: 10.1097/MPG.0000000000002598, PMID: 31880664

[29] Patil S Ananthan A Nanavati R Nataraj G Prasad P. Effect of different methods of pasteurization on bactericidal action of human milk: a prospective observational study. Indian J Med Res. (2019) 150:504–7. doi: 10.4103/ijmr.IJMR_600_18, PMID: 31939395 PMC6977360

[30] Escuder-Vieco D Rodríguez JM Espinosa-Martos I Corzo N Montilla A García-Serrano A. High-temperature short-time and holder pasteurization of donor milk: impact on milk composition. Life. (2021) 11:114. doi: 10.3390/life11020114, PMID: 33546253 PMC7913308

[31] Müller K Toll LJ Manzardo OA Baumgartner J Nickel E Wenzel F. Holder pasteurization: comparison of water-bath and dry-tempering devices. Front Pediatr. (2022) 10:879853. doi: 10.3389/fped.2022.879853, PMID: 35874591 PMC9301034

[32] Nebbia S Giribaldi M Cavallarin L Bertino E Coscia A Briard-Bion V. Differential impact of holder and high temperature short time pasteurization on the dynamic in vitro digestion of human milk in a preterm newborn model. Food Chem. (2020) 328:127126. doi: 10.1016/j.foodchem.2020.127126, PMID: 32492605

[33] Giribaldi M Nebbia S Briard-Bion V Jardin J Peila C Coscia A. Simulated dynamic digestion reveals different peptide releases from human milk processed by means of holder or high temperature-short time pasteurization. Food Chem. (2022) 369:130998:130998. doi: 10.1016/j.foodchem.2021.130998, PMID: 34507088

[34] Hassinger D Clausen DM Nitka S Herdt A Griffin I. Analysis of Disialyllacto-N-Tetraose (DSLNT) content in milk from mothers of preterm infants. J Hum Lact. (2020) 36:291–8. doi: 10.1177/0890334420904041, PMID: 32109186

[35] Giribaldi M Coscia A Peila C Antoniazzi S Lamberti C Ortoffi M. Pasteurization of human milk by a benchtop high-temperature short-time device. Innov Food Sci Emerg Tech. (2016) 36:228–33. doi: 10.1016/j.ifset.2016.07.004

[36] Mallardi D Piemontese P Liotto N Colombo RM Dodaro A Schiavello A. New operating approach to limit *Bacillus cereus* contamination of donor human milk. J Hum Lact. (2022) 38:102–7. doi: 10.1177/08903344211002563, PMID: 33745375

[37] Moran L Rowe MT Hagan JA. The effect of various heat activation treatments on fast, intermediate and slow germinating spores of *Bacillus* spp. Lett Appl Microbiol. (1990) 10:43–6. doi: 10.1111/j.1472-765X.1990.tb00091.x

[38] Ranieri ML Huck JR Sonnen M Barbano DM Boor KJ. High temperature, short time pasteurization temperatures inversely affect bacterial numbers during refrigerated storage of pasteurized fluid milk. J Dairy Sci. (2009) 92:4823–32. doi: 10.3168/jds.2009-2144, PMID: 19762797

[39] Escuder-Vieco D Espinosa-Martos I Rodríguez JM Fernández L Pallás-Alonso CR. Effect of HTST and holder pasteurization on the concentration of immunoglobulins, growth factors, and hormones in donor human milk. Front Immunol. (2018) 9:2222. doi: 10.3389/fimmu.2018.02222, PMID: 30319659 PMC6170621

[40] Klotz D Schreiner M Falcone V Jonas D Kunze M Weber A. High-temperature short-time treatment of human milk for bacterial count reduction. Front Pediatr. (2018) 6:359. doi: 10.3389/fped.2018.00359, PMID: 30538974 PMC6277678

[41] Donalisio M Rittà M Francese R Civra A Tonetto P Coscia A. High temperature-short time pasteurization has a lower impact on the antiviral properties of human milk than holder pasteurization. Front Pediatr. (2018) 6:304. doi: 10.3389/fped.2018.00304, PMID: 30460212 PMC6232822

[42] Dussault N Cayer MP Landry P de Grandmont MJ Cloutier M Thibault L. Comparison of the effect of holder pasteurization and high pressure processing on human milk bacterial load and bioactive factors preservation. Pediatr Gastroenterol Nutr. (2021) 72:756–62. doi: 10.1097/MPG.0000000000003065, PMID: 33847290 PMC8549451

[43] Irazusta A Rodríguez-Camejo C Jorcin S Puyol A Fazio L Arias F. High-pressure homogenization and high hydrostatic pressure processing of human milk: preservation of immunological components for human milk banks. J Dairy Sci. (2020) 103:5978–91. doi: 10.3168/jds.2019-17569, PMID: 32418693

[44] Pitino MA Unger S Doyen A Pouliot Y Aufreiter S Stone D. High hydrostatic pressure processing better preserves the nutrient and bioactive compound composition of human donor milk. J Nutr. (2019) 149:497–504. doi: 10.1093/jn/nxy302, PMID: 30770541 PMC6398389

[45] Jarzynka S Strom K Barbarska O Pawlikowska E Minkiewicz-Zochniak A Rosiak E. Combination of high-pressure processing and freeze-drying as the most effective techniques in maintaining biological values and microbiological safety of donor milk. Int J Environ Res Public Health. (2021) 18:2147. doi: 10.3390/ijerph18042147, PMID: 33671763 PMC7926441

[46] Barbarska O Strom K Oledzka G Calvo J Gayà A López-Mendoza MC. Effect of nonthermal processing on human milk bactericidal activity against *Escherichia coli*. J Pediatr Gastroenterol Nutr. (2020) 70:864–7. doi: 10.1097/MPG.0000000000002673, PMID: 32443049

[47] Kothari A Pitino MA Unger S Perreault V Doyen A Pouliot Y. Preservation of anti-cytomegalovirus activity in human milk following high pressure processing compared to holder pasteurization. Front Nutr. (2022) 19:918814. doi: 10.3389/fnut.2022.918814, PMID: 35662924 PMC9160983

[48] Pitino MA Unger S Gill A McGeer AJ Doyen A Pouliot Y. High pressure processing inactivates human cytomegalovirus and hepatitis a virus while preserving macronutrients and native lactoferrin in human milk. Innov Food Sci Emerg Technol. (2022) 75:102891. doi: 10.1016/j.ifset.2021.102891

[49] Marousez L Sprenger N De Lamballerie M Jaramillo-Ortiz S Tran L Micours E. High hydrostatic pressure processing of human milk preserves milk oligosaccharides and avoids formation of Maillard reaction products. J Clin Nutr. (2022) 41:1–8. doi: 10.1016/j.clnu.2021.11.013, PMID: 34861623

[50] Zhang J Lee NA Duley JA Cowley DM Shaw PN Bansal N. Comparing the effects of hydrostatic high-pressure processing vs holder pasteurisation on the microbial, biochemical and digestion properties of donor human milk. Food Chem. (2022) 373:131545. doi: 10.1016/j.foodchem.2021.131545. Epub 2021 Nov 8, PMID: 34839967

[51] Wesolowska A Brys J Barbarska O Strom K Szymanska-Majchrzak J Karzel K. Lipid profile, lipase bioactivity, and lipophilic antioxidant content in high pressure processed donor human milk. Nutrients. (2019) 11:1972. doi: 10.3390/nu11091972, PMID: 31438647 PMC6770840

[52] Pitino MA Alashmali SM Hopperton KE Unger S Pouliot Y Doyen A. Oxylipin concentration, but not fatty acid composition, is altered in human donor milk pasteurised using both thermal and non-thermal techniques. Br J Nutr. (2019) 122:47–55. doi: 10.1017/S0007114519000916, PMID: 31006410

[53] Koh J Victor AF Howell ML Yeo JG Qu Y Selover B. Bile salt-stimulated lipase activity in donor breast milk influenced by pasteurization techniques. Front Nutr. (2020) 7:552362. doi: 10.3389/fnut.2020.552362, PMID: 33282897 PMC7689290

[54] Wemelle E Marousez L Lesage J De Lamballerie M Knauf C Carneiro L. In Vivo assessment of antioxidant potential of human milk treated by holder pasteurization or high hydrostatic pressure processing: a preliminary study on intestinal and hepatic markers in adult mice. Antioxidants. (2022) 11:1091. doi: 10.3390/antiox11061091, PMID: 35739988 PMC9220199

[55] Wemelle E Marousez L de Lamballerie M Knauf C Lesage J. High hydrostatic pressure processing of human milk increases Apelin and GLP-1 contents to modulate Gut contraction and glucose metabolism in mice compared to holder pasteurization. J Nutrients. (2022) 14:219. doi: 10.3390/nu14010219, PMID: 35011094 PMC8747192

[56] Smyczynska U Bartlomiejczyk MA Stanczak MM Sztromwasser P Wesolowska A Barbarska O. Impact of processing method on donated human breast milk microRNA content. PLoS One. (2020) 15:e0236126. doi: 10.1371/journal.pone.0236126 32667939 PMC7363072

[57] Marousez L Tran L Micours E De Lamballerie M Gottrand F Pierrat V. Metabolic hormones in human breast milk are preserved by high hydrostatic pressure processing but reduced by holder pasteurization. J Food Chem. (2022) 30:131957. doi: 10.1016/j.foodchem.2021.131957 34990954

[58] Rocha-Pimienta J Martillanes S Ramírez R Garcia-Parra J Delgado-Adamez J. *Bacillus cereus* spores and *Staphylococcus aureus* sub. aureus vegetative cells inactivation in human milk by high-pressure processing. Food Control. (2020) 113:107212. doi: 10.1016/j.foodcont.2020.107212

[59] Consales A Cerasani J Sorrentino G Morniroli D Colombo L Mosca F. The hidden universe of human milk microbiome: origin, composition, determinants, role, and future perspectives. Eur J Pediatr. (2022) 181:1811–20. doi: 10.1007/s00431-022-04383-1, PMID: 35124754 PMC9056486

[60] Zhang J Duley JA Cowley DM Shaw PN Koorts P Bansal N. Comparative proteomic analysis of donor human milk pasteurized by hydrostatic high-pressure. Food Chem. (2023) 1:134264. doi: 10.1016/j.foodchem.2022.134264 36182857

[61] Nash DP Jonas J. Structure of pressure-assisted cold denatured lysozyme and comparison with lysozyme folding intermediates. Biochemistry. (1997) 36:14375–83. doi: 10.1021/bi970881v, PMID: 9398155

[62] Wejryd E Martí M Marchini G Werme A Jonsson B Landberg E. Low diversity of human milk oligosaccharides is associated with necrotising Enterocolitis in extremely low birth weight infants. Nutrients. (2018) 10:1556. doi: 10.3390/nu10101556, PMID: 30347801 PMC6213888

[63] Underwood MA. Probiotics and the prevention of necrotizing enterocolitis. J Pediatr Surg. (2019) 54:405–12. doi: 10.1016/j.jpedsurg.2018.08.055, PMID: 30241961

[64] Ye A Anema SG Singh H. High-pressure–induced interactions between milk fat globule membrane proteins and skim milk proteins in whole milk. J Dairy Sci. (2004) 87:4013–22. doi: 10.3168/jds.S0022-0302(04)73542-0, PMID: 15545361

[65] Black EP Setlow P Hocking AD Stewart CM Kelly AL Hoover DG. Response of spores to highpressure processing. Inst Food Technol. (2007) 6:113–9. doi: 10.1111/j.1541-4337.2007.00021.x

[66] Wuytack EY Boven S Michiels CW. Comparative study of pressureinduced germination of *Bacillus subtilis* spores at low and high pressures. Appl Environ Microbiol. (1998) 64:3220–4. doi: 10.1128/AEM.64.9.3220-3224.1998, PMID: 9726863 PMC106713

[67] Wuytack EY Soons J Poschet F Michiels CW. Comparative study of pressureand nutrient-induced germination of *Bacillus subtilis* spores. Appl Environ Microbiol. (2000) 66:257–61. doi: 10.1128/AEM.66.1.257-261.2000, PMID: 10618233 PMC91815

[68] Clouston JG Wills PA. Initiation of germination and inactivation of *Bacillus pumilus* spores by hydrostatic pressure. J Bacteriol. (1969) 97:684–90. doi: 10.1128/jb.97.2.684-690.1969, PMID: 5773022 PMC249746

[69] Van Opstal I Vanmuysen SC Wuytack EY Michiels CW. Inactivation of *Bacillus cereus* spores in milk by mild pressure and heat treatments. Commun Agric Appl Biol Sci. (2003) 68:7–10. PMID: 14702650

[70] Demazeau G Plumecocq A Lehours P Martin P Couëdelo L Billeaud C. A new high hydrostatic pressure process to assure the microbial safety of human milk while preserving the biological activity of its main components. Front Public Health. (2018) 6:306. doi: 10.3389/fpubh.2018.00306, PMID: 30460221 PMC6232532

[71] Sterilization of health care products – general requirements for characterization of a sterilizing agent and the development, validation and routine control of a sterilization process for medical devices. (2009). Available at: https://www.iso.org/obp/ui#iso:std:iso:14937:ed-2:v1:en.

[72] Alexandre-Gouabau M-C Moyon T Cariou V Antignac J-P Qannari EM Croyal M. Breast milk lipidome is associated with early growth trajectory in preterm infants. Nutrients. (2018) 10:164. doi: 10.3390/nu10020164, PMID: 29385065 PMC5852740

[73] Wesolowska A Sinkiewicz-Darol E Barbarska O Strom K Rutkowska M Karzel K. New achievements in high-pressure processing to preserve human milk bioactivity. Front Pediatr. (2018) 6:323. doi: 10.3389/fped.2018.00323 30519550 PMC6250976

[74] Almutawif Y Hartmann B Lloyd M Lai CT Rea A Geddes D. *Staphylococcus aureus* enterotoxin production in raw, holder-pasteurized, and ultraviolet-C-treated donated human milk. Breastfeed Med. (2019) 14:262–70. doi: 10.1089/bfm.2018.0217, PMID: 30817174

[75] Papademas P Mousikos P Aspri M. Optimization of uv-c processing of donkey milk: an alternative to pasteurization? Animals. (2021) 11:1–10. doi: 10.3390/ani11010042, PMID: 33379250 PMC7824723

[76] Blázquez E Rodríguez C Ródenas J Navarro N Rosell R Pina-Pedrero S. UV-C irradiation is able to inactivate pathogens found in commercially collected porcine plasma as demonstrated by swine bioassay. Vet Microbiol. (2019) 239:108450. doi: 10.1016/j.vetmic.2019.108450, PMID: 31753544 PMC7130610

[77] Blázquez E Rodríguez C Ródenas J Navarro N Riquelme C Rosell R. Evaluation of the effectiveness of the surepure turbulator ultraviolet-C irradiation equipment on inactivation of different enveloped and non-enveloped viruses inoculated in commercially collected liquid animal plasma. PLoS One. (2019) 14:e0212332. doi: 10.1371/journal.pone.0212332 30789926 PMC6383881

[78] Colás-Medà P Nicolau-Lapeña I Viñas I Neggazi I Alegre I. Bacterial spore inactivation in orange juice and orange peel by ultraviolet-C light. Food Secur. (2021) 10:855. doi: 10.3390/foods10040855, PMID: 33920777 PMC8103511

[79] Assal N Boone R Harris RA Gabriel M Sasges M Petri B. Inactivation of group I and group II *Clostridium botulinum* spores by ultraviolet irradiation in water. Int J Food Microbiol. (2023) 395:110191. doi: 10.1016/j.ijfoodmicro.2023.110191, PMID: 37019040

[80] Pendyala B Patras A Gopisetty VVS Sasges M Balamurugan S. Inactivation of bacillus and clostridium spores in coconut water by ultraviolet light. Foodborne Pathog Dis. (2019) 16:704–11. doi: 10.1089/fpd.2019.2623, PMID: 31135181

[81] Abe H Endo K Shiba M Niibe Y Miyata S Satake M. Flow path system of ultraviolet C irradiation from xenon flash to reduce bacteria survival in platelet products containing a platelet additive solution. Transfusion. (2020) 60:1050–9. doi: 10.1111/trf.15757, PMID: 32187695

[82] Baysal AH Molva C Unluturk S. UV-C light inactivation andnmodeling kinetics of *Alicyclobacillus acidoterrestris* spores in white grape and apple juices. Int J Food Microbiol. (2013) 166:494–8. doi: 10.1016/j.ijfoodmicro.2013.08.015, PMID: 24042001

[83] Sigman M Burke KI Swarner OW Shavlik GW. Effects of microwaving human milk: changes in IgA content and bacterial count. J Am Diet Assoc. (1989) 89:690–2. doi: 10.1016/S0002-8223(21)02229-X, PMID: 2723294

[84] Quan R Yang C Rubinstein S Lewiston NJ Sunshine P Stevenson DK. Effects of microwave radiation on anti-infective factors in human milk. Pediatrics. (2010) 89:667–9. 1557249

[85] Levchenko A Lukoyanova O Borovik T Levchenko M Sevostianov D Sadchikov P. The novel technique of microwave heating of infant formulas and human milk with direct temperature monitoring. J Biol Regul Homeost Agents. (2017) 31:353–7. PMID: 28685536

[86] Leite JAS Migotto AMA Landgraf M Quintal VS Gut JAW Tadini CC. Pasteurization efficiency of donor human milk processed by microwave heating. LWT. (2019) 115:108466. doi: 10.1016/j.lwt.2019.108466

[87] Leite JAS Robinson RC Salcedo J Ract JNR Quintal VS Tadini CC. The effect of microwave-assisted heating on bioactive and immunological compounds in donor human milk. LWT. (2022) 161:113306. doi: 10.1016/j.lwt.2022.113306

[88] Martysiak-Żurowska D Puta M Kielbratowska B. The effect of convective heating and microwave heating on antioxidant enzymes in pooled mature human milk. Int Dairy J. (2019) 91:41–7. doi: 10.1016/j.idairyj.2018.12.008

[89] Yoshida Y Azuma M Furukawa K Mizuno K Den H Kamiya T. Microwave heating of human milk with direct temperature monitoring. J Hum Lact. (2022) 38:323–31. doi: 10.1177/08903344211047452, PMID: 34704491

[90] Martysiak-Żurowska D Malinowska-Pańczyk E Orzołek M Kusznierewicz B Kiełbratowska B. Effect of microwave and convection heating on selected nutrients of human milk. Food Chem. (2022) 1:130958. doi: 10.1016/j.foodchem.2021.130958 34479011

[91] Martysiak-Żurowska D Puta M Kiełbratowska B Wesołowska A. Neurotrophic factors in human milk in early lactation and the effect of holder and microwave pasteurization on their concentrations. J Pediatr Gastroenterol Nutr. (2021) 72:900–5. doi: 10.1097/MPG.0000000000003071, PMID: 33976087

[92] Mikawa T Mizuno K Tanaka K Kohda C Ishii Y Yamamoto K. Microwave treatment of breast milk for prevention of cytomegalovirus infection. Pediatr Int. (2019) 61:1227–31. doi: 10.1111/ped.13954, PMID: 31282599

[93] Malinowska-Pańczyk E Królik K Skorupska K Puta M Martysiak-Żurowska D Kiełbratowska B. Microwave heat treatment application to pasteurization of human milk. Innov Food Sci Emerg Technol. (2019) 52:42–8. doi: 10.1016/j.ifset.2018.11.005

[94] Blackshaw K Wu J Proschogo N Davies J Oldfield D Schindeler A. The effect of thermal pasteurization, freeze-drying, and gamma irradiation on donor human milk. Food Chem. (2022) 373:131402. doi: 10.1016/j.foodchem.2021.131402, PMID: 34741965

[95] Blackshaw K Wu J Valtchev P Lau E Banati RB Dehghani F. The effects of thermal pasteurisation, freeze-drying, and gamma-irradiation on the antibacterial properties of donor human milk. Food Secur. (2021) 10:2077. doi: 10.3390/foods10092077, PMID: 34574186 PMC8469727

[96] Lee SY Park SH Kang DH. Inactivation of *Alicyclobacillus acidoterrestris* spores in apple and orange juice concentrates by gamma irradiation. J Food Prot. (2014) 77:339–44. doi: 10.4315/0362-028X.JFP-13-133, PMID: 24490932

[97] Dauphin LA Newton BR Rasmussen MV Meyer RF Bowen MD. Gamma irradiation can be used to inactivate *Bacillus anthracis* spores without compromising the sensitivity of diagnostic assays. Appl Environ Microbiol. (2008) 74:4427–33. doi: 10.1128/AEM.00557-08, PMID: 18515484 PMC2493171

[98] Arnold LD. Currents in human milk banking. The Lactariums of France, part 1 the Lactarium Docteur Raymond Fourcade in Marmande. J Hum Lact. (1994) 10:125–6. doi: 10.1177/089033449401000221, PMID: 7619254

[99] Oliveira MM Aragon DC Bomfim VS Trevilato TMB Alves LG Heck AR. Development of a human milk concentrate with human milk lyophilizate for feeding very low birth weight preterm infants: a preclinical experimental study. PLoS One. (2019) 14:e0210999. doi: 10.1371/journal.pone.0210999, PMID: 30785913 PMC6382113

[100] Martysiak-Zurowska D Rozek P Puta M. The effect of freeze-drying and storage on lysozyme activity, lactoferrin content, superoxide dismutase activity, total antioxidant capacity and fatty acid profile of freeze-dried human milk. Dry Technol. (2020) 40:615–25. doi: 10.1080/07373937.2020.1824188

[101] Hahn W-H Bae SP Song S Park S Lee J Seo J-B. The freeze-drying does not influence the proteomic profiles of human milk. J Matern Fetal Neonatal Med. (2020) 33:2069–74. doi: 10.1080/14767058.2018.1538349 30418097

[102] Salcedo J Gormaz M Lopez-Mendoza MC Nogarotto E Silvestre D. Human milk bactericidal properties: effect of lyophilization and relation to maternal factors and milk components. J Pediatr Gastroenterol Nutr. (2015) 60:527–32. doi: 10.1097/MPG.0000000000000641, PMID: 25406523

[103] Garcia ML Burgos J Sanz B Ordoñez JA. Effect of heat and ultrasonic waves on the survival of two strains of *Bacillus subtilis*. J Appl Bacteriol. (1989 Dec) 67:619–28. doi: 10.1111/j.1365-2672.1989.tb02535.x, PMID: 2515184

[104] Salerno T Siqueira AK Pinto JPAN Cunha MLRSD Silvestre PK Condas LAZ. Safety issues of raw milk: evaluation of bacteriological and physicochemical characteristics of human milk from a bank in a teaching hospital, focusing on Staphylococcus species. Rev Inst Med Trop São Paulo. (2021) 63:e54. doi: 10.1590/s1678-9946202163054, PMID: 34231819 PMC8266376

[105] Dewitte C Courdent P Charlet C Dumoulin D Courcol R Pierrat V. Contamination du lait maternel par Une flore aérobie: évaluation des pertes pour un lactarium [contamination of human milk with aerobic flora: evaluation of losses for a human milk bank]. Arch Pediatr. (2015) 22:461–7. doi: 10.1016/j.arcped.2015.02.011, PMID: 25858450

[106] Widger J O'Connell NH Stack T. Breast milk causing neonatal sepsis and death. Clin Microbiol Infect. (2010) 16:1796–8. doi: 10.1111/j.1469-0691.2010.03071.x, PMID: 19832716

[107] Jawa G Hussain Z da Silva O. Recurrent late-onset group B streptococcus sepsis in a preterm infant acquired by expressed breastmilk transmission: a case report. Breastfeed Med. (2013) 8:134–6. doi: 10.1089/bfm.2012.0016, PMID: 23373437

[108] Behari P Englund J Alcasid G Garcia-Houchins S Weber SG. Transmission of methicillin-resistant *Staphylococcus aureus* to preterm infants through breast milk. Infect Control Hosp Epidemiol. (2004) 25:778–80. doi: 10.1086/502476, PMID: 15484804

[109] Taylor JMW Sutherland AD Aidoo KE Logan NA. Heat-stable toxin production by strains of *Bacillus cereus*, *Bacillus firmus*, *Bacillus megaterium*, Bacillus simplex and *Bacillus licheniformis*. FEMS Microbiol Lett. (2005) 242:313–7. doi: 10.1016/j.femsle.2004.11.022, PMID: 15621453

[110] Lotte R Chevalier A Boyer L Ruimy R. *Bacillus cereus* invasive infections in preterm neonates: an up-to-date review of the literature. Clin Microbiol Rev. (2022) 35:e0008821. doi: 10.1128/cmr.00088-21, PMID: 35138121 PMC8826972

[111] Lewin A Quach C Rigourd V Picaud JC Perreault T Frange P. *Bacillus cereus* infection in neonates and the absence of evidence for the role of banked human milk: case reports and literature review. Infect Control Hosp Epidemiol. (2019) 40:787–93. doi: 10.1017/ice.2019.110, PMID: 31172903

[112] Polidori P Vincenzetti S. Effects of thermal treatments on donkey milk nutritional characteristics. Recent Pat Food Nutr Agric. (2013) 5:182–7. doi: 10.2174/2212798405666131224104541, PMID: 24365337

